# Paternal outcrossing success differs among faba bean genotypes and impacts breeding of synthetic cultivars

**DOI:** 10.1007/s00122-021-03832-z

**Published:** 2021-05-07

**Authors:** Lisa Brünjes, Wolfgang Link

**Affiliations:** Plant Breeding Methodology, Department of Crop Sciences, Georg-August Universität Göttingen, Carl-Sprengel-Weg 1, 37075 Göttingen, Germany

## Abstract

**Key message:**

Faba bean genotypes showed significant and marked genetic differences in their success as pollen donors to cross-fertilized seeds. The findings may improve exploitation of heterosis in synthetic cultivars.

**Abstract:**

In partially allogamous crops such as faba bean (*Vicia faba* L.), increasing the share of heterosis in a synthetic cultivar can improve yield and yield stability. The share of heterosis in such synthetic cultivars is increased by higher degrees of cross-fertilization. This trait is defined as percentage of cross-fertilized seeds among all seeds and is a crucial parameter in breeders’ yield predictions. Current approaches use degree of cross-fertilization to predict inbreeding and share of heterosis, they even consider genotype-specific degrees; yet, all genotypes are assumed to contribute equally to the cross-fertilized seeds. Here, we expect faba bean genotypes to differ in their success rates as pollen donors, i.e. in paternal outcrossing success. To quantify the variation of both, the degree of cross-fertilization and the paternal outcrossing success, we assessed these parameters in inbred lines and F1 hybrids, grown in four polycrosses composed of eight genotypes each. We identified the paternal genotype of 500 to 800 seeds per genotype and polycross using SNP markers. In both traits, we found marked and significant variation among inbred lines and among F1 hybrids, as well as between inbred lines and F1. Based on our findings, we discuss how differential paternal outcrossing success influences the amount of inbreeding in synthetic cultivars. Our findings offer the potential for a better management and exploitation of heterotic yield increase in faba bean.

**Supplementary Information:**

The online version contains supplementary material available at 10.1007/s00122-021-03832-z.

## Introduction

Pollen-dispersing plants do not have the ability to exercise choice of their mating partners, in contrast to most animals. Individual plants produce pollen that is transferred by wind, insects or other animals and may successfully fertilize many plants at the same time. Similarly, under open-pollination, maternal plants of cross-fertilizing species generally bear offspring sired by more than one pollen-donor plant. Differences in paternal outcrossing success, i.e. differential success rates as pollen donors to initiate seed set are common in outcrossing plants (Marshall et al. 2000; Bernasconi 2003; Torimaru et al. 2012; Pannell and Labouche 2013). Genetic differences underlying this trait are relevant in breeding of synthetic cultivars of non-autogamous plants, such as clover (*Trifolium repens*, *Trifolium pratense*), alfalfa (*Medicago sativa*), sunflower (*Helianthus annuus*), oilseed rape (*Brassica napus*) and grasses (*Lolium perenne*, *Lolium multiflorum*), but also for open-pollinated varieties of crops such as maize (*Zea mais*) and rye (*Secale cereal*e) developed for organic farming (Messmer et al. 2015). Here, we focus on the paternal outcrossing success in faba bean (*Vicia faba* L.), a partially allogamous grain legume whose degree of cross-fertilization varies strongly around a mean of about 50%, depending on genotype and on environmental conditions (Link 1990; Link and Ederer 1993; Suso et al. 1999, 2001, 2008a; Gasim et al. 2004). Faba bean is pollinated by wild and domestic bees. Without bee pollination, the yield is decreased by more than 30% (Bishop and Nakagawa 2021). Both self-pollen and cross-pollen are fertile and fully compatible (Link and Ghaouti 2012).

Current faba bean cultivars are typically synthetic cultivars (synthetics) that have been shown to outperform inbred lines in grain yield due to the partially expressed heterosis in such synthetics (Ghaouti and Link 2009). Full heterosis and near-maximum selection intensity would be realized in hybrid cultivars. However, the production of hybrid cultivars requires pollination control based on, e.g., a stable CMS system. This is not available in faba bean (Link 2006; Maalouf et al. 2019). A considerably high level of heterosis for grain yield was found for faba bean: 27–76% in spring faba beans (Stelling et al. 1994) and 33–51% in winter beans (Link et al. 1994b). Since hybrid cultivars are not available, synthetics are produced as second-best choice. Synthetic cultivars are developed by selecting a limited number of superior inbred lines as components and by subsequent production of seed from a mixed stand of these lines. This seed is produced by open pollination in spatial isolation (Smith 2004; Becker 2019). The generation in which the parental components are mixed is called Syn-0. The consecutive multiplication generations result from natural pollination and without further selection. They are called Syn-1, Syn-2, Syn-3, Syn-4, the latter typically represents the seed sold to farmers. In synthetic cultivars, plants are more or less heterozygous and heterosis enhances their performance. The realized heterosis will permanently stay in such a synthetic population, thereby effectuating an increased yield compared to the yield of Syn-0.

The choice of lines and their share in the Syn-0 population govern the yield of the synthetic. However, the large number of possible Syn-0 populations renders field trials of all promising synthetics unfeasible. With, for instance, 50 candidate lines as components and with sets of four components to be selected per synthetic, a total of 230,000 different sets can be created. Thus, one crucial task in breeding of synthetic cultivars is to predict the most promising synthetic, i.e. the optimal set of inbred lines, without field-testing any of these possible sets. Several approaches to predict the performance of synthetics based on characteristics of their inbred line components have been developed (Kinman und Sprague 1945; Wright 1973; Becker 1982, 2019; Gallais 1990; Link und Ederer 1993; Kutka und Smith 2007). In case of a partially allogamous crop such as faba bean, these approaches may include the level of cross-fertilization of the inbred lines (Geiger 1982; Ederer und Link 1992; Link et al. 1994a). Higher cross-fertilization leads to higher heterozygosity and thus to a higher share of heterosis, which results in higher yield.

The population-genetic make-up of a partially allogamous synthetic is expected to change across the initial steps of creation and propagation. For completely allogamous crops and if the Syn-1 is created by a fully random mating mixture of components, Hardy–Weinberg equilibrium for each locus is reached after only one generation of open pollination. From Syn-1 onwards and under ideal conditions (e.g. absence of epistasis and drift), such populations stay at a stable level of heterozygosity and performance (Becker 1982). In contrast, in a synthetic of a partially allogamous crop, the yield potential is expected to further increase in the generations following Syn-1, as it takes several successive generations of partially allogamous propagation until the inbreeding coefficient reaches its minimum and therefore the yield level will then stay constant (Link et al. 1994a; Kelly et al. 2000). With partial allogamy, each population following Syn-0 is composed of plants with diverse inbreeding coefficients $\mathrm{F}$. This mode of reproduction leads to a complex genetic composition of such populations, rendering the yield of partially allogamous synthetic candidate cultivars difficult to predict.

Currently, only approaches for approximate yield prediction of generation Syn-1 are available (Link and Ederer 1993). These approaches already take a genotype-specific value for the degree of cross-fertilization into account. Yet, so far it has to be assumed that all components in Syn-0 contribute the same amount of pollen to the cross-fertilized seeds. However, in reality we expect differences between these genotypes in their success rates as pollen donors when siring viable seeds via cross-fertilization.

**Fig. 1:**
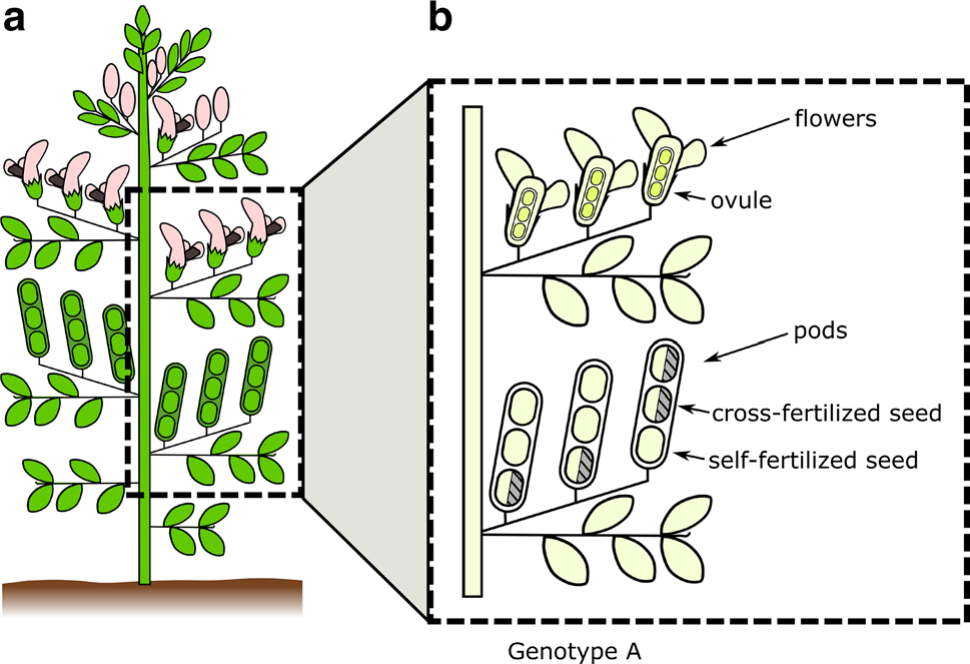
Schematic representation of the partial allogamy in faba bean. From a flowering plant (**a**), a section is shown in further detail (**b**): the ovules inside the flowers, ready for fertilization (top), flowers already developed into pods (bottom). The completely white seeds in these pods illustrate self-fertilized seeds. Seeds that are half white and half-filled illustrate cross-fertilized seeds. In this example, the genotype has a degree of cross-fertilization of 4/9, i.e. about 44%

There are several possible origins of pollen that lead to viable seeds. Following the pathways of pollen dispersal from pollen grain production to successful ovule fertilization (Harder and Wilson 1998; Minaar et al. 2019), an ovule in a faba bean flower can receive pollen in four different ways, (i) autogamous selfing, i.e. pollen from the same flower, (ii) geitonogamous selfing, i.e. pollen transmitted from another flower of the same plant, (iii) intra-genotype cross-fertilization, i.e. pollen transmitted from a flower of a different plant of the same genotype, which is genetically indistinguishable from a selfing event, (iv) inter-genotype cross-fertilization, i.e. pollen from a flower of a different genotype (Bond and Poulsen 1983). Even though geitonogamous selfing, intra-genotype cross-fertilization and inter-genotype cross-fertilization are all the result of cross-flower pollen transport by pollen vectors, only inter-genotype cross-fertilization results in cross-fertilization in a genetic sense. Not every successfully fertilized ovule develops into a mature seed (Bond and Poulsen 1983; Link and Stützel 1995). Accordingly, the degree of cross-fertilization C is defined as the share of viable seeds of a genotype that paternally does not descend from this genotype, given that flowering takes place under natural access of pollinators (Link et al. 1994a; Metz et al. 1994). Here, a genotype is considered in its role as a maternal plant bearing the seeds. The term self-fertilization is used in a genetic sense and does include autogamous and geitonogamous self-fertilization as well as intra-genotype cross-fertilization (see Fig. 1).

**Fig. 2:**
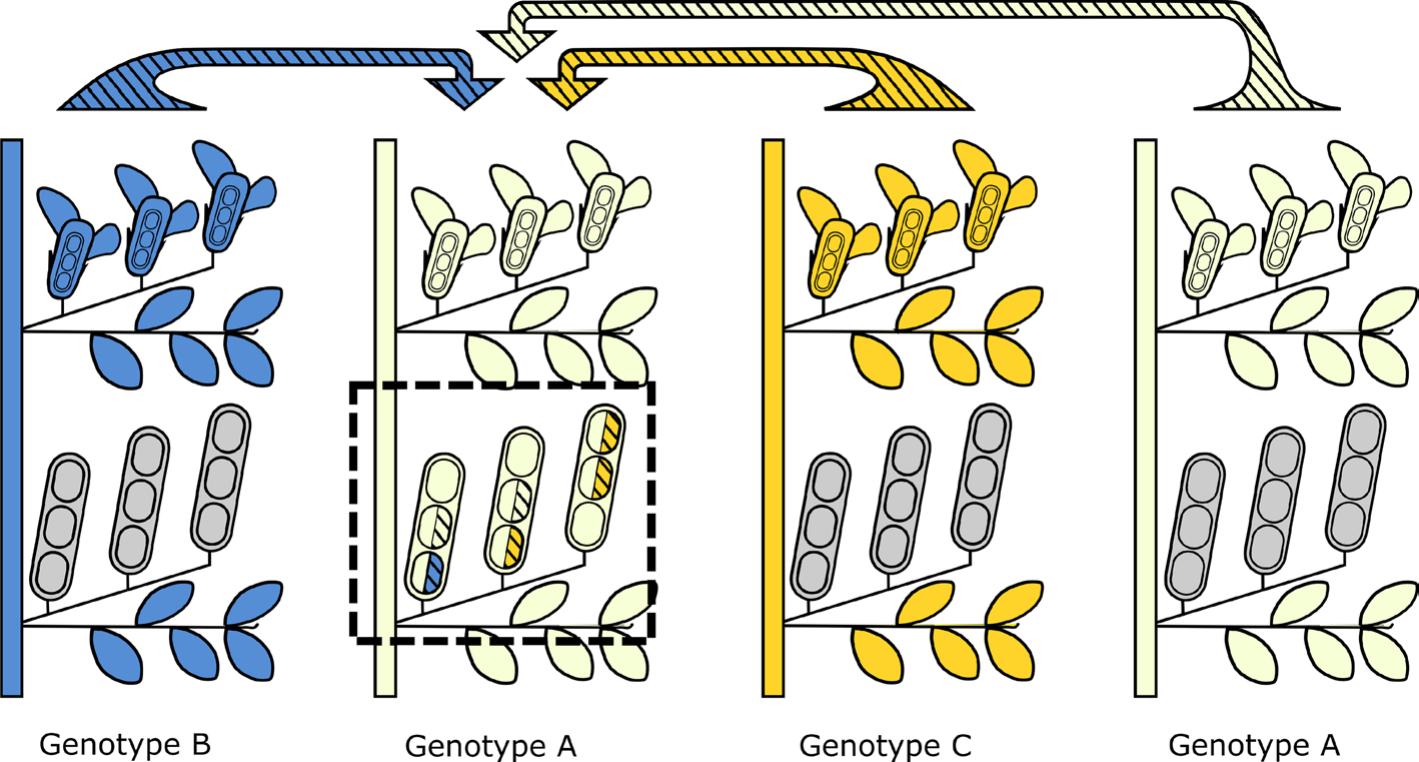
Schematic representation of paternal outcrossing success in faba bean. Genotype A (compare Fig. 1) is shown with its seeds (black frame), focussing on the composition of the cross-fertilized seeds. The striped halves of the seeds indicate cross-fertilization by three different pollen donors (arrows from genotypes A, B and C). Four seeds of genotype A originate from inter-genotype cross-fertilization: one from genotype B and three from genotype C. In this example, genotype C has a higher paternal outcrossing success than genotype B. In addition, two seeds originate from intra-genotype cross-fertilization (with the same genotype A as female and male parent). The three white seeds of genotype A originate from self-fertilization

The paternal outcrossing success P of a genotype is based on the combined share of intra-genotype and inter-genotype cross-fertilization and can therefore not easily be measured by just evaluating the genotypes of the offspring. We follow here the concept and terminology of Barret (2003) and Riday et al. (2013) (but see, for alternative concepts and terms, these authors: Apsit et al. 1989; Kohn and Barrett 1994; Kaufman et al. 1998; Fishman 2000; Skogsmyr and Lankinen 2002; Bernasconi 2003; Delph and Herlihy 2011; Torimaru et al. 2012; Pannell and Labouche 2013; McCallum and Chang 2016; Minaar et al. 2019). In our work, the paternal outcrossing success describes the success of a genotype to produce viable offspring by cross-fertilizing the ovules of other plants. We consider a genotype in its role as paternal plant or pollen donor to cross-fertilized seeds (see Fig. 2).

Any genetic or environmental variation of cross-fertilization in a current generation influences the level of inbreeding of the next generation and consequently yield. The genetic variation in paternal outcrossing success further increases the variation in inbreeding between candidate synthetics, which was neglected so far. In previous studies, it was assumed that cross-pollen originates in equal doses from all individuals of the population (Busbice 1969, 1970; Wright 1977; Geiger 1982; Link and Ederer 1993). However, studies on faba bean found different numbers of pollen grains produced by different genotypes, differential pollen viability, or differential attractiveness of flowers for pollinators (Kambal et al. 1976; Suso et al. 2008b; Chen 2009; Gasim und Link 2009; Bailes et al. 2018).

Given such variation in reproductive traits, we expect marked variation in the paternal outcrossing success. Furthermore, we know that inbred plants allow more foreign pollen to fertilize their ovules than non-inbred plants (e.g. F1 hybrids) (Drayner 1959; Ebmeyer 1988; Link et al. 1994a). We expect that heterosis contributes to the extent of paternal outcrossing success, which would result in a larger paternal outcrossing success of non-inbred plants compared to inbred plants. Here, we compared the paternal outcrossing success of highly inbred and non-inbred plants which has not been discussed or studied so far. If, within a population, the degrees of cross-fertilization are small, differences in paternal outcrossing success do not contribute much to changes in the level of inbreeding of the next generation. However, with large degrees of cross-fertilization, a clearly unequal paternal contribution to cross-fertilized seeds markedly increases inbreeding in the following generation. Thus, we first estimated the degrees of cross-fertilization of the faba bean genotypes used in our field trials. Second, we experimentally quantified the paternal outcrossing success and challenged the assumption that the components of Syn-0 contribute equally as pollen donors to the subsequent generation.

For the degree of cross-fertilization C, we tested the following hypotheses:

- Faba bean genotypes differ markedly and significantly in C.
- F1 hybrids have a smaller C than inbred lines.

For the paternal outcrossing success P, we tested the following hypotheses:

- The genotypes differ markedly and significantly in P.
- F1 hybrids have a larger P than inbred lines.
- The size of P depends on the genotypes of both mating partners: the maternal plant and the pollen donor.

We expected high C and low P values for inbred lines versus low C and high P values for F1 hybrids. We further tested the hypothesis that C and P values are negatively associated within a group of inbred lines, i.e. at a constant level of inbreeding.

## Material and methods

### Plant material and study sites

To estimate the degree of cross-fertilization $\mathrm{C}$ and the paternal outcrossing success $\mathrm{P}$, we grew 16 faba bean genotypes in field trials. The plant material consisted of 12 winter faba bean inbred lines and four F1 hybrids, derived from the ‘Göttingen Winter Bean Population’, which is a genetically wide winter bean population founded in Göttingen, Germany in 1989 (Link and Arbaoui 2006). The used winter bean genotypes set flowers and seed even if sown in spring, albeit some days later than spring types.

The inbred lines were selected based on their distinguishability with few SNP markers. Inbred lines were further selected based on similar initiation of flowering (max. difference two days) to optimize the possibility of cross-fertilization, and other trait expressions such as high winter survival after autumn-sowing, little lodging and high disease tolerance. Data on these traits were collected during previous years of field trials (unpublished field books, Göttingen).

The inbred lines were developed via single seed descent for at least eight generations. The F1 hybrids were developed via manual crossing of eight of those inbred lines. Each field trial was composed of eight genotypes grown in a polycross design. A polycross is a combined cultivation of different genotypes in a spatial design that allows random cross-pollination (Melchinger et al. 2008). Each genotype included in a polycross serves as pollen receptor (maternal role) and pollen donor (paternal role) at the same time. In each polycross, each genotype was included with 64 individual plants.

The polycrosses were grown in the surroundings of Göttingen, Germany, each in spatial isolation from other faba bean fields to prevent contamination with foreign faba bean pollen. We used three genetic polycross sets (set 0, set A, and set B). While set 0 consisted of 8 inbred lines, sets A and B consisted of six inbred lines and two F1 hybrids (Table 1). While the polycrosses of sets 0 and A were sown in spring, the polycross of set B was sown in October, before winter (see Supplementary Table 1).

**Table 1:** Inbred lines and F1-hybrids used in our studies (X = included in the corresponding set, – = not included)

Genotype*	Set 0	Set A	Set B
S_046	X	X	–
S_085	X	X	X
F1(S_046 x S_085)	–	–	X
S_199	–	X	–
Fam157	X	X	–
F1(S_199 x Fam157)	–	–	X
S_019	X	–	X
S_035	X	–	X
F1(S_019 x S_035)	–	X	–
S_025	X	–	X
S_217	X	–	X
F1(S_025 x S_217)	–	X	–
S_120	X	–	–
S_145	–	X	–
S_235	–	X	–
S_003	–	–	X

*F1-genotypes: Reciprocal crosses were not differentiated and both reciprocal cross directions were used in unspecified dose

F1 hybrids of faba bean realize lower degrees of cross-fertilization than inbred lines (Drayner 1956, 1959; Link 1990). Here, F1 hybrids were included to investigate the influence of the level of inbreeding on C and P. The inbred parental lines of the F1 hybrids in a polycross were not included in the same polycross, because this would render the assessment of paternity more complicated. Instead, they were included as inbred lines in the other set and vice versa, with inbred line S_085 in set B as one exception from this rule (see Table 1). In total, we collected data from 16 genotypes: 12 inbred lines and four F1 hybrids.

To achieve a high validity of our results, we grew the three sets of genotypes in a total of four polycrosses at three locations in three years in the surroundings of Göttingen. We aimed at having environmental variation in the activity, abundance and species diversity of bee pollinators which directly impacts the degree of cross-fertilization (Marzinzig et al. 2018).

The obtained data include trials at these four environments (i.e. combinations of years and locations): One polycross with set 0 (2014, location Garteschänke, GAR), two polycrosses with set A (2015 and 2016, location Dragoneranger, DRA), and one with set B (2016, location Deppoldshausen, DEP). Each polycross consisted of eight blocks with eight single plants of each genotype per block, resulting in 64 single plants of each genotype across the whole polycross and a total of 512 plants (see Supplementary Fig. 1). The direct and diagonal plant neighbourhood between different genotypes was completely balanced when considering the total of eight blocks (Morgan 1988; Fleck und Ruckenbauer 1989, see Supplementary Fig 2).

We sowed the 512 single plants manually and kept record of all individuals. Sowing density was 15 plants m^−2^. Plants were sown in double rows with 22 cm distance between rows and 22 cm distance within rows. The distance between double rows was 40 cm to allow access of persons. In addition, we sowed several adjacent rows with these same genotypes as reserve, in order to replace any non-emerged or sick plant (see Supplementary Fig. 3). Later, at the begin of flowering, the adjacent rows were removed.

### Handling of plants during flowering

Asynchronous flowering leads to a bias in P because the plants that flower simultaneously have a higher chance of exchanging pollen than asynchronously flowering plants. To minimize this bias, on a day when plants of all genotypes in the polycross were flowering, i.e. when concurrence of full bloom (CFB) occurred, we labelled all single plants. On each tiller, we attached a blue label to the inflorescence that was in full blossom (see Supplementary Fig. 4, Supplementary Table 1). Flowering occurred under natural access of pollinators. We did not carry out observations on the abundance of pollinators or their foraging behaviour but regard the site-specific pollinator population as part of the environmental effect. At maturity, we manually and individually harvested all plants allowing us to assign the maternal genotype and the corresponding field data to each bag of harvested seeds.

### Identification of pollen donors

We employed SNP markers to conduct paternity testing and to identify the pollen donors (i.e. paternal genotype) of a large number of seeds. Aiming to test the paternity of about 770 seeds of each genotype and polycross, we chose about 12 seeds from each of the 512 single plants for SNP analysis. We selected those pods that grew at the node marked with the blue label, as these pods developed from flowers that were open at a day when nearly all other plants in the trial were flowering (97.9% to 99.8% in four polycrosses, see Supplementary Table 1).

Selected pods were analysed with all their seeds. These seeds were re-sown in the greenhouse and the seedlings were genotyped with SNP markers (Ellwood et al. 2008; Cottage et al. 2012; Webb et al. 2015; O'Sullivan, personal information), using the KASP method (Robinson and Home 2011; Semagn et al. 2014).

To identify pollen donors, a selection of SNP markers was chosen for each set. We identified three (four) SNP markers as the minimal number of markers needed for the unambiguous identification of pollen donors for set B (sets 0 and A). For validation, we chose a second selection of four markers that allowed unambiguous identification for each set. This approach resulted in seven SNP markers used for set B and eight markers used for sets 0 and A to identify the pollen donors. Further, the selection of SNPs was chosen such that also the two F1 hybrids in set A and set B were homozygous at those specific SNPs. Thus, the algorithms for the paternity test were the same for inbred lines as for F1 hybrids. To confirm that the manually produced F1 genotypes in sets A and B were indeed hybrids, we used two more SNP markers in those two sets (i.e. in total ten and nine SNPs in sets A and B).

For each seedling, the marker data were used to confirm the maternal genotype and to identify the paternal genotype. To that aim, we first identified for each SNP, which DNA base belonged to the maternal genotype, attributing the other base to the paternal genotype. If none of the two bases of any SNP could be attributed to the maternal genotype, the marker data were omitted, and the seedling was resampled. If also the resampled marker data did not fully match the maternal genotype, the seedling was omitted. Next, for each SNP, the base attributed to the paternal genotype was compared with the corresponding bases of the eight possible fathers. If, for all SNPs, these bases fully matched the bases of one of the possible fathers, that genotype was accepted as father. If there was no complete match, iteratively, each single base was ignored and if the remaining bases matched a unique genotype, the genotype was accepted. If again no father could be identified, iteratively pairs of bases were ignored, and a unique match of the remaining bases with a genotype was accepted. All seedlings where no father was identified in this way were omitted. This algorithm was implemented in Python.

The identified paternal and maternal genotypes could either be the same, resulting in a seed being classified as self-fertilized, or different (cross-fertilization). For each polycross, we sampled between 5460 and 6429 offspring plants and were able to identify the paternal genotype in 97.9–99.5% of these plants. Each of the eight genotypes of a polycross was included in the following statistical analyses with 572–839 offspring plants and their identified maternal and paternal genotypes (Supplementary Tables 2–5). After determining the maternal and paternal genotype of each sampled seed, we calculated for each genotype and block of each set (i) the degree of cross-fertilization C and (ii) the paternal outcrossing success P.

In the polycross of set B, instead of sowing inbred line S_120 as intended, inbred line S_085 had been sown, which is the paternal line of one of the hybrids included in the same polycross. To differentiate between S_085 and F1(S_046 x S_085) as pollen donors in set B, we employed two additional SNP markers.

### Statistical analyses

#### Estimation of C

The degree of cross-fertilization C is the share of inter-genotype cross-fertilized seeds among all seeds. For the estimation of C, we first created a dataset where we block-wise joined all seeds and their SNP-derived results from the eight plants of a maternal genotype into one unit of analysis.

The statistical analysis was performed using R (version 3.4.2, R Core Team 2017). We employed a generalized linear model (GLM) with a binomial distribution and logit link function (package “MASS”, Venables and Ripley 2002) to analyse the effect of maternal genotype on the degree of cross-fertilization. The response variable was a matrix with two columns, where the first column was the number of cross-fertilized seeds and the second column was the number of self-fertilized seeds. Thereby, we accounted for differences in seed sample size. Maternal genotype and block were included as fixed effects. Set A (location DRA) was analysed across two years. Here, block was nested within year and the interaction of year and maternal genotype was included in the model. It was checked if residuals were normally distributed, and the model was tested for potential overdispersion of data (Crawley 2013).

#### Estimation of P

Here, the aim is to estimate the paternal outcrossing success P for each genotype, based on our SNP data. First, for all unequal pairs of paternal and maternal genotypes, the number of cross-fertilized seeds of the maternal genotype sired by the paternal genotype was counted. In contrast, for equal pairs, the SNP marker results are identical for autogamous and geitonogamous self-fertilization on one hand and intra-genotype cross-fertilization on the other hand. Therefore, our method is not able to distinguish between intra-genotype cross-fertilized seeds and self-fertilized seeds. For each equal pair, we assumed that the number of seeds sired by intra-genotype cross-fertilization is identical to the mean of the numbers of inter-genotype cross-fertilized seeds realized by the other paternal genotypes on the same maternal genotype. Consequently, in our field trials with eight genotypes, for each equal pair of paternal and maternal genotype, we set the number of intra-genotype cross-fertilized seeds to the mean of the number of seeds paternally cross-fertilized by the seven other genotypes. Thereby, we increased the number of cross-fertilized seeds for each maternal genotype by 1/7, resulting in 1/8 intra-genotype cross-fertilized seeds and 7/8 inter-genotype cross-fertilized seeds for each maternal genotype. Thus, the paternal outcrossing success of a genotype on itself is 1/8; therefore, the maternal genotype does not influence P.

To estimate P, we calculated GLMs with a binomial distribution and a logit link function to account for the different sample sizes (package “MASS”, Venables and Ripley 2002). The response variable in the analysis of P was a matrix where the first column was the number of cross-fertilized seeds for each pair of maternal and paternal genotypes. The second column was the number of all cross-fertilized seeds of the same maternal genotype minus the number in the first column. Maternal genotype, paternal genotype and their two-way interactions were treated as fixed effects because they were not randomly chosen, rather they were chosen based on similar onset of flowering and distinguishability with SNP markers. In set A, year was also included as a fixed effect. We checked if the residuals were normally distributed and tested the model for potential overdispersion of data (Crawley 2013).

#### Post-hoc tests

We used the logit link function for the GLMs and all statistical tests and calculations were performed on the logit scale. Only for data visualisation, we used the back-transformed values, calculated employing the inverse logit function (package “boot”, Canty and Ripley 2019).

We computed least square mean values of C and P (package “emmeans”, Lenth et al. 2019) to account for the different variances between the genotypes. To compute likelihood ratio ${\chi }^{2}$-tests, we applied the Anova function from the package “car” (Fox and Weisberg 2019). First, the differences between the group of inbred lines and the group of F1 hybrids with respect to their C and P values were tested for significance. Second, the differences of genotypes within these groups were tested. Here, we conducted multiple comparisons of least square means with user-defined contrasts (package “multcomp”, Hothorn et al. 2008) and employed the Bonferroni correction to adjust *p* values for multiple testing. The visualisation was done employing the “ggplot2” package (Wickham 2016) to create plots and the “multcompView” package (Piepho 2004) to produce compact letter displays.

## Results

### Degree of cross-fertilization

To test if the selected 16 faba bean genotypes (12 inbred lines and four F1 hybrids) differ in their degree of cross-fertilization C, we grouped them in three sets (0, A, and B) of eight genotypes each. Set 0 only consisted of inbred lines, while sets A and B each consisted of two F1 hybrids and six inbred lines. The genotypes of sets 0 and B were both evaluated in field polycrosses that were grown in one year and at one location. The genotypes of set A were grown in polycrosses at one location in two subsequent years. For a restricted number of seeds per plant harvested from the polycrosses, we identified the maternal and paternal genotypes using SNP markers. We then employed generalized linear models (GLMs) to estimate C of each genotype.

The results of the GLMs indicate that the maternal genotypes had a significant effect on degrees of cross-fertilization (Table 2). In sets A and B, we found a significant effect of blocks. Since set A was grown in two years, we included the year and the year × maternal genotype interactions in the model. Both model terms turned out to have significant effects on C.

**Table 2:** Analysis of deviance table for generalized linear models fitted to estimate the degrees of cross-fertilization of sets 0, A, and B

Model terms	df**	Set 0		Set A		Set B	
		Deviance	*p* value	Deviance	*p* value	Deviance	*p* value
Null	63; (120)	380.88		1090.71		825.08	
Maternal genotype (MG)	7; (7)	303.11	< 0.001	488.27	< 0.001	690.03	< 0.001
Block	7; (14)	4.63	0.705	83.76	< 0.001	32.96	< 0.001
Year*	(1)	–	–	255.77	< 0.001	–	–
Year × MG*	(7)	–	–	54.34	< 0.001	–	–
Residual	49; (98)	73.14		208.58		102.09	

The model terms were added sequentially. The columns show the degrees of freedom corresponding to the additional model term, the resulting change in deviance, and the *p* value when likelihood-ratio chi-squared tests are used to test for significance. Sets 0 and B were grown in one year at one location. Set A was grown in two years at one location

* Applies only to set A. ** Numbers on the left show degrees of freedom in sets 0 and B, while numbers in brackets show degrees of freedom in set A

**Fig. 3:**
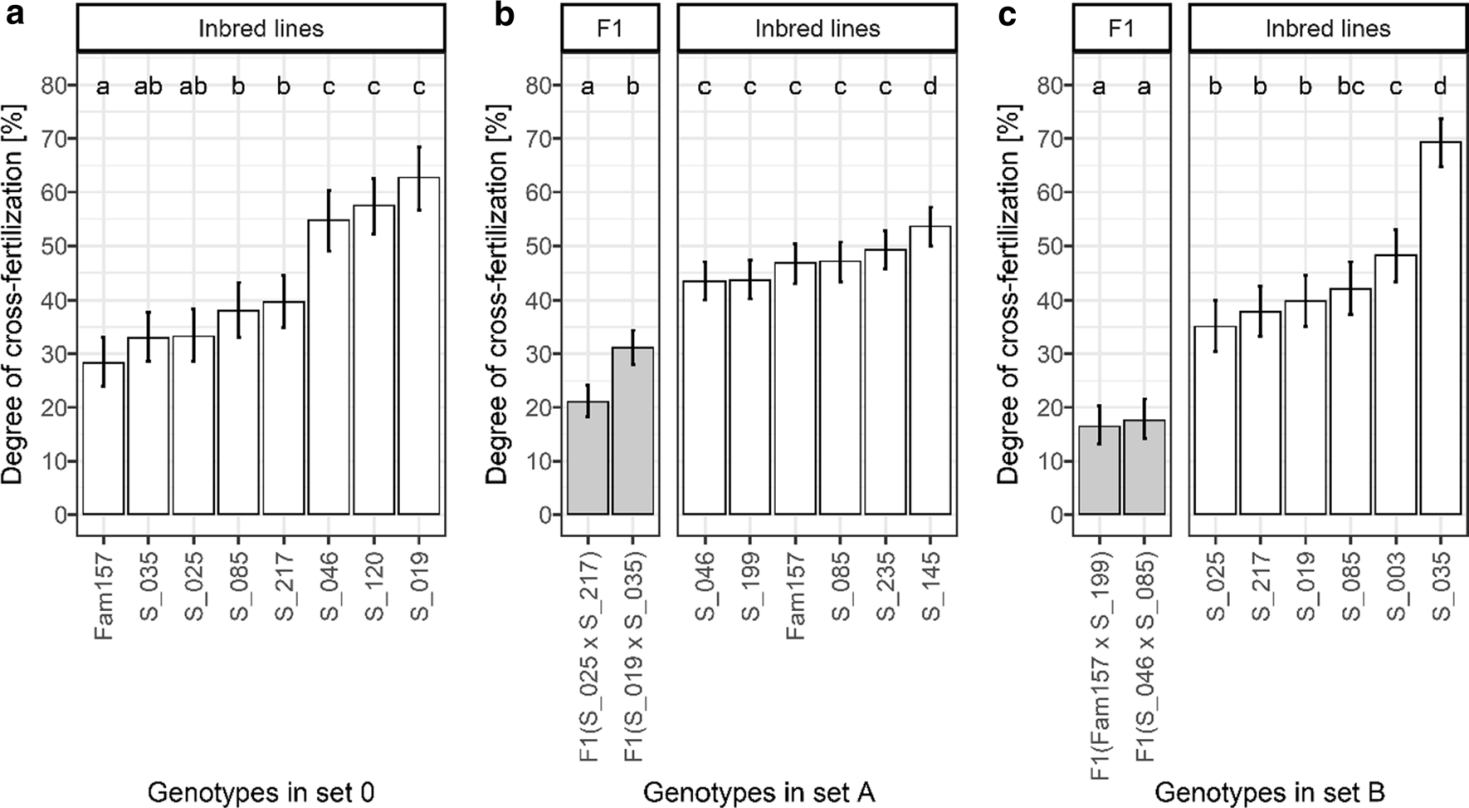
Degree of cross-fertilization of the faba bean genotypes in different sets of genotypes: **a** set 0 in one year (2014) and at one location (GAR), **b** set A in two years (2015, 2016) and at one location (DRA), and **c** set B in one year (2016) and at one location (DEP). For each set, different letters show significant differences among least square means (*p* = 0.05, multiple comparisons of means with user-defined contrasts and Bonferroni correction). Vertical lines show 95-percent confidence intervals. Least square mean values for each environment (combinations of year × location) are shown in Supplementary Table 6

The 16 genotypes showed a high variation of the degree of cross-fertilization (Fig. 3). In set 0 which consisted of inbred lines, we found marked and significant differences in C between the eight inbred lines with means of C ranging from 28.3 to 62.5% (Fig. 3a). In set A which consisted of F1 hybrids and inbred lines, we also found high variation of C (Fig. 3b), with lower C values for the hybrids (21% and 31%) and higher values for the inbred lines (44–54%). Both within the group of the two F1 as well as within the group of the six inbred lines, we found significant differences between genotypes. The degree of cross-fertilization of the F1 hybrids was significantly lower than that of the inbred lines (mean of F1 hybrids: 26%; mean of inbred lines: 47%; *p* < 0.001, multiple comparisons of means with user-defined contrasts and Bonferroni correction). The lower degree of cross-fertilization of the F1 hybrids was observed in both years. The mean C values of the F1 hybrids were 19% and 35% in the years 2015 and 2016, respectively. For the inbred lines, the respective mean C values were 41% and 54%. In set B, we also found lower C values for the F1 hybrids (16% and 18%) compared to the inbred lines (35–69%, Fig. 3c). While the difference between the two F1 hybrids was not significant, several of the inbred lines differed markedly and significantly in C. As in set A, the mean C value of the F1 hybrids was significantly lower than that of the inbred lines (mean of F1 hybrids: 17%; mean of inbred lines: 45%; *p* < 0.001, multiple comparisons of means with user-defined contrasts and Bonferroni correction).

### Paternal outcrossing success

The paternal outcrossing success P was estimated for the 16 faba bean genotypes grown in the same four polycrosses used to estimate the degree of cross-fertilization C. In each polycross, eight genotypes competed with one another for paternal outcrossing success.

Each plant produced a certain share of self-fertilized seeds determined by the degree of cross-fertilization. The remaining seeds are the result of paternal outcrossing from other plants. For each block and all plants of each genotype, the sum of all seeds originating from paternal outcrossing was for our analyses set to 100%, i.e. the sum of the cross-fertilized seeds on each maternal genotype corresponds to 100%. This sum is shared among the possible pollen donors and this share is the paternal outcrossing success P of each genotype. Accordingly, the average paternal outcrossing success of the eight pollen donors is one eighth, i.e. 12.5%. This average is the same on each of the eight maternal genotypes, and thus there must be zero difference between maternal genotypes for average paternal outcrossing success. The same is true for differences due to blocks and—in set A—due to the two years. After identifying the paternal genotypes of the seeds using SNP markers, we used GLMs to estimate P of each genotype.

The results of the GLMs showed that the paternal genotype and the paternal genotype × maternal genotype interaction had significant effects on the paternal outcrossing success (Table 3). As described above, there was no variation for main effects of maternal genotype and of blocks. Since set A was grown in two years, we included the year main-effect, the two-way interactions of year × paternal genotype and year × maternal genotype, as well as the three-way interaction paternal genotype × maternal genotype × year in the model. Also, as expected, year had no effect on P and the interaction of maternal genotype × year was almost absent. However, the paternal genotype × year and the paternal genotype × maternal genotype × year interactions turned out to have significant effects on P. In Supplementary Tables 8–15, we present the values for P and the corresponding interaction effect of each PG × MG combination.

**Table 3:** Analysis of deviance table for generalized linear models fitted to analyse the paternal outcrossing success of Sets 0, A and B

Model terms	df**	Set 0		Set A		Set B	
		Deviance	*p* value	Deviance	*p* value	Deviance	*p* value
Null	511; (1023)	959		3317		1653	
Paternal genotype (PG)	7; (7)	189	< 0.001	1376	< 0.001	741	< 0.001
Maternal genotype (MG)	7; (7)	0	1	0	1	0	1
Year*	(1)	–	–	0	1	–	–
PG × MG	49; (49)	182	< 0.001	440	< 0.001	307	< 0.001
PG × Year	(7)	–	–	118	< 0.001	–	–
MG × Year*	(7)	–	–	< 1	1	–	–
Block (within Year*)	7; (14)	0	1	0	1	0	1
PG × MG × Year	(49)	–	–	124	< 0.001	–	–
Residual	441; (882)	587		1258		605	

The model terms were added sequentially. The columns show the degrees of freedom corresponding to the additional model term, the resulting change in deviance, and the *p* value when likelihood-ratio chi-squared tests are used to test for significance. Sets 0 and B were grown in one year at one location. Set A was grown in two years at one location

*Applies only to set A. **Numbers on the left show degrees of freedom in sets 0 and B, while numbers in brackets show degrees of freedom in set A

**Fig. 4:**
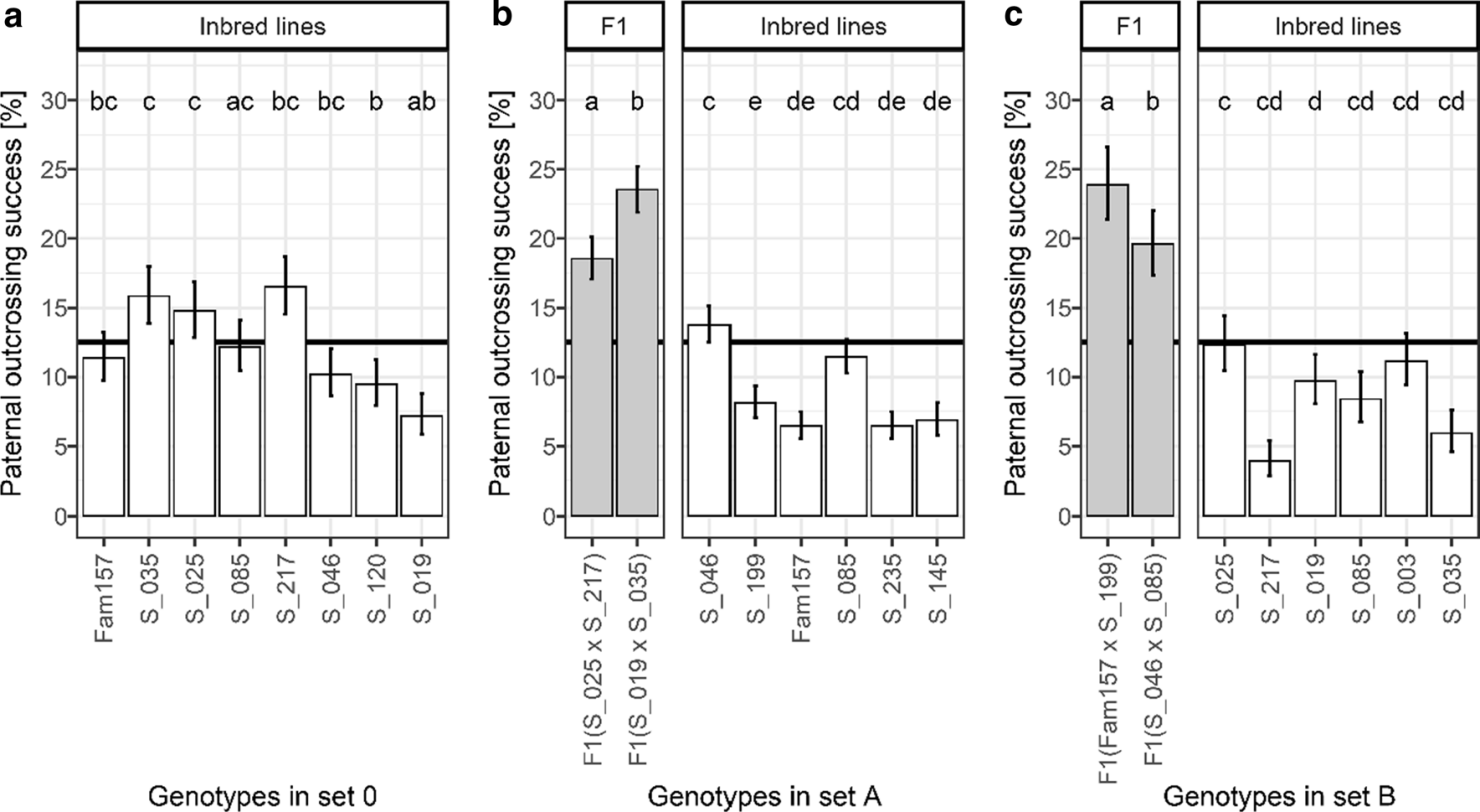
Paternal outcrossing success of the faba bean genotypes in different sets of genotypes: **a** set 0 in one year (2014) and at one location (GAR), **b** set A in two years (2015, 2016) and at one location (DRA), and **c** set B in one year (2016) and at one location (DEP), sorted by mean degree of cross-fertilization of the same set. For each set, different letters show significant differences among least square means (*p* = 0.05, multiple comparisons of means with user-defined contrasts and Bonferroni correction). Vertical lines show 95-percent confidence intervals. The horizontal line indicates a paternal outcrossing success of 12.5%. Least square mean values for each environment (combinations of year × location) are shown in Supplementary Table 7

The 16 faba bean genotypes showed a high variation of the paternal outcrossing success (Fig. 4). Note that the genotypes in Fig. 4 are sorted by their mean degrees of cross-fertilization in ascending order (compare with Fig. 3).

In set 0, we found marked and significant differences in P between several inbred lines. For some inbred lines, P was more than twice as high when compared to other lines, with mean values of P ranging from 7.2 to 16.5% (Fig. 4a).

In set A, we likewise found a high variation of P (Fig. 4b) with significant differences between genotypes both within the group of the two F1 hybrids and within the group of the six inbred lines. Further, the two F1 hybrids had markedly higher P values (18.5% and 23.5%) than the six inbred lines (6.4–13.8%). The mean value of the F1 hybrids was significantly larger than that of the inbred lines (mean of F1 hybrids: 20.9%; mean of six inbred lines: 8.5%; *p* < 0.001, multiple comparisons of means with user-defined contrasts and Bonferroni correction). The observation of higher paternal outcrossing success of the F1 hybrids was found in both years independently. In 2015, the mean P values of the F1 hybrids and inbred lines were 27.7% and 8.2%, respectively, while in 2016 these values were 20.2% and 8.8%. Thus, the observed higher trait expression of the F1 hybrids compared to the inbred lines was larger in the first year than in the second year. This difference is highlighted by the significant paternal genotype × year interaction in Table 3.

In set B, we also found higher P values for the F1 hybrids (19.6% and 23.9%) compared to the inbred lines (3.9–12.3%, Fig. 4c). In the group of F1 hybrids and in the group of inbred lines, we found significant differences in P between genotypes. The mean paternal outcrossing success of the F1 hybrids was markedly and significantly larger than of the mean of the inbred lines (F1 hybrids: 21.7%; inbred lines: 8.6%; *p* < 0.001). When comparing sets A and B, we found similar mean $\mathrm{P}$ values for inbred lines (8.5% and 8.0% in sets A and B, respectively) and for F1 hybrids (20.9% and 21.7%). The difference between the two groups was more pronounced in set B.

Across all four polycrosses, the paternal outcrossing success varied between 3.9 and 16.5% in inbred lines, and between 16.2 and 24.9% in F1 hybrids (Supplementary Table 7). As all genotypes included in one polycross compete against each other for paternal outcrossing success, we would expect, given more extensive sampling, that the mean P value of all genotypes approaches 12.5%. Here, the realized mean values for the different polycrosses are between 11.9 and 12.2% (Supplementary Table 7, bottom row). However, the P values of the individual genotypes are markedly different from these mean values.

### Correlation of C and P

**Fig. 5:**
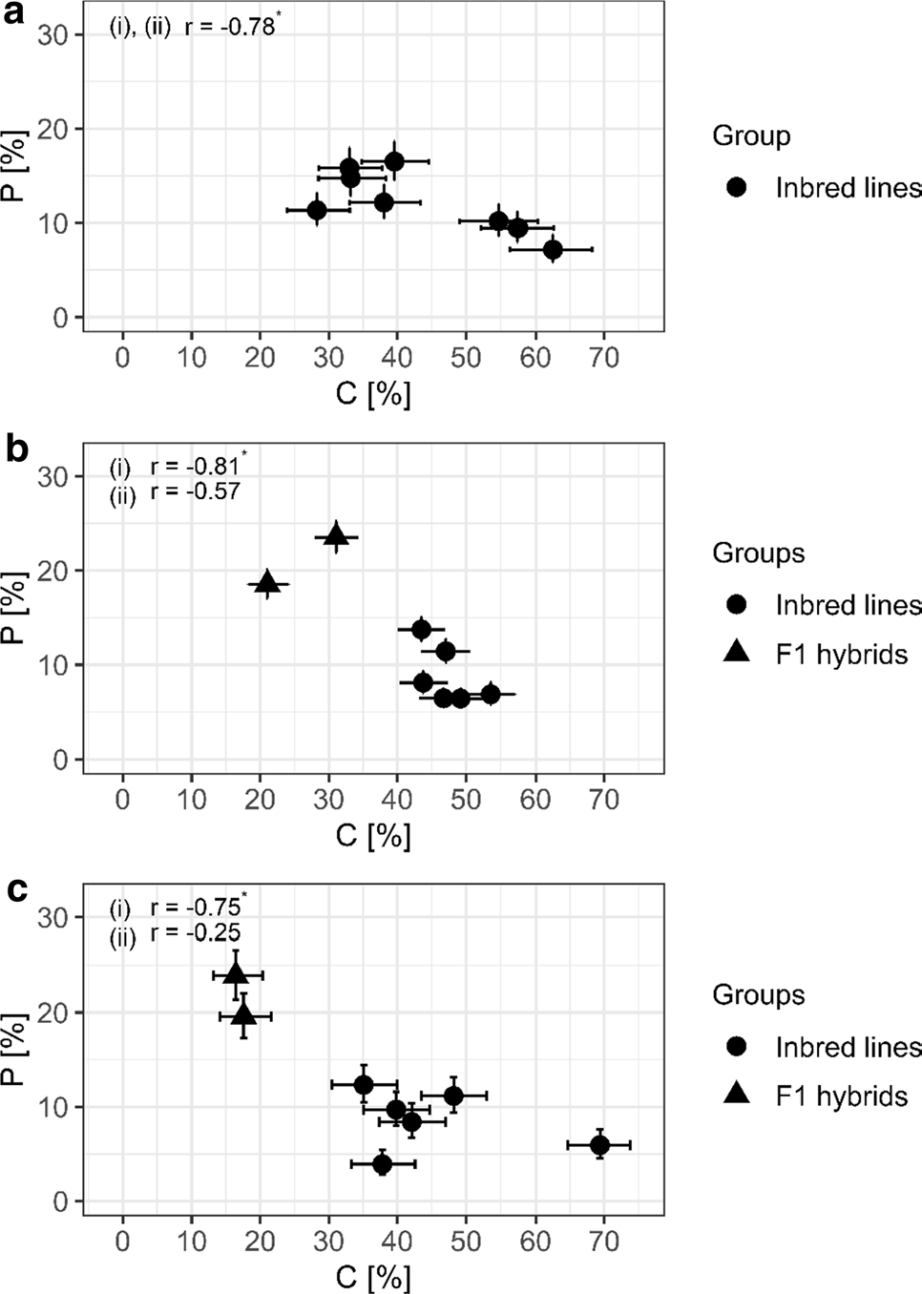
Correlation of degree of cross-fertilization C and paternal outcrossing success P in different sets of genotypes: **a** set 0 in one year (2014) and at one location (GAR), **b** set A in two years (2015, 2016) and at one location (DRA), and **c** set B in one year (2016) and at one location (DEP). Error bars show 95-percent confidence intervals. Correlation coefficients are calculated based on Pearson's product-moment correlation coefficient for (i) all genotypes, (ii) only inbred lines

To test if C and P values are negatively correlated within the group of inbred lines, we employed Pearson's product-moment correlation test (Fig. 5). Indeed, for set 0 which only contained inbred lines, we found a significant negative correlation between both traits with a high absolute value of the correlation coefficient (*r* = − 0.78, *p* value = 0.02, see Fig. 5a). However, in sets A and B which also included F1 hybrids, the correlation for the inbred lines between both traits was medium and non-significant (set A: *r* = − 0.57, *p* value = 0.24, see Fig. 5b; set B: *r* = − 0.25, *p* value  = 0.63, see Fig. 5c). Only when the F1 hybrids were included, we found a high and significant negative correlation between both traits (set A: *r* = − 0.81, *p* value  = 0.01, see Fig. 5b; set B: *r* = − 0.75, *p* value  = 0.03, see Fig. 5c).

## Discussion

### Field trial

The purpose of the field trial design was to control some of the non-genetic effects that might influence the pollen distribution among genotypes. With an isochronic anthesis and a balanced neighbourhood across genotypes, the conditions should be near to ideal such that each genotype had an equal basic opportunity, with respect to neighbourhood and proximity, to be pollen parent of the seeds of any other genotype. However, pollen dispersal between inflorescences and plants depends on the small-scaled foraging behaviour of the pollinators, which has barely been studied so far (as an exception, see Brunet et al. 2019 on alfalfa). The effect of distance between plants or genotypes on gene dispersal or on degree of cross-fertilization has barely been studied for faba bean (*cf*. Link and von Kittlitz 1989). Meanwhile, floral traits, such as floral design and display, have been shown to have a strong influence on the level of cross-fertilization. In particular, the number of flowers and inflorescences, as well as the sugar concentration of the nectar have effects on a plant’s attractiveness to pollinators (Suso et al. 2005, 2008b; Palmer et al. 2009; Bailes et al. 2018), which might affect the movement of pollinators between single plants.

We did neither assess nor control the composition and abundances of pollinators. Foraging bees in faba bean were shown to express a species-specific foraging behaviour (pollination, nectar robbing, visiting extrafloral nectaries). The amount of seed set and the rate of effectuated cross-fertilization after one pollination visit seem to depend on the pollinator species (Marzinzig et al. 2018). Given these differences between pollinator species, our results are probably influenced by the specific abundances and composition of pollinators at a given location. We regard the site-specific pollinator fauna as part of the environmental impact on the outcomes.

### Degree of cross-fertilization and heterosis

We found marked variation in the degrees of cross-fertilization C between the studied genotypes. Specifically, there were significant differences between degrees of cross-fertilization of (i) the two F1 hybrids included in a set, (ii) the six or eight inbred lines included in a set, and (iii) the two F1 hybrids as one group and the six inbred lines as another group.

The degree of cross-fertilization of some of the inbred lines differed strikingly among the sets and environments (location and year). For example, the degree of cross-fertilization C of line S_019 was 63% in set 0 (GAR 2014), while it was only 40% in set B (DEP 2016). Line S_035 showed the opposite trend, with 33% in set 0 (GAR 2014) and 70% in set B (DEP 2016). Line Fam157 also responded strongly to the environment ranging from 28% in Set 0 (GAR 2014) and 36–58% in set A (DRA 2015 and 2016, respectively). The trait expression of genotypes is markedly influenced by both the environment and the companion faba bean genotypes, as well as by the genotype-environment interaction (see Table 2). In set A, we found for all genotypes higher values for C in the year 2016 than in 2015 (see Supplementary Table 6). Bishop et al. (2017) found that heat stress may lead to an increased level of cross-fertilization. In 2016, during the flowering of the plants, the polycross of set A was exposed to a period of high temperatures, which might explain the higher values for C in that year.

The F1 hybrids tended to have a lower degree of cross-fertilization than their parents (Supplementary Table 6). For each set and environment, the two F1 hybrids consistently had the lowest value for C of all genotypes. The effect of inbreeding level on the degree of cross-fertilization has already been discussed by Drayner (1959) who studied three different F1 hybrids as well as partly inbred genotypes (F2–F6) of English winter beans, employing hilum colour as morphological marker to detect cross-fertilization. She found values for C of 4%, 7% and 21% for the hybrids, and their mean was significantly lower than the mean of any of the more inbred generations. This was supported by Link (1990), who found the degree of cross-fertilization to vary from 7 to 82% in German spring beans along with a similar trend of increasing values for C with a decrease in heterozygosity, comparing the F1, F2 and F3 generation and the parental inbred lines. Similarly, Link et al. (1994a) reported a degree of cross-fertilization from 36 up to 82% in eight inbred lines of German spring beans, and in the F1-generation only 7–56% and thus much lower values. Suso et al. (2008a) studied faba bean populations grown under different agro-ecological conditions in Europe and estimated their multi-locus outcrossing rate as well as the respective inbreeding coefficient of maternal plants. They found outcrossing rates from 14 to 72% for different populations and agro-ecological conditions. Their further results suggest a negative heterotic component of cross-fertilization, thereby supporting our findings.

Our findings on German winter beans are therefore in line with previous observations and strongly support the notion of negative heterosis for the degree of cross-fertilization. Accordingly, heterosis is positive for the amount of selfing: non-inbred plants have higher degrees of self-fertilization than inbred lines. The effect of the level of inbreeding on the degree of cross-fertilization, to our knowledge, has not been studied in mixed mating species other than faba bean.

### Paternal outcrossing success and heterosis

The paternal outcrossing success P is the result of the competition of the paternal plants for the cross-fertilized seeds produced by the maternal plant. This competition takes place in several stages of seed development starting from the pollen production and ending with the mature seed. Here, we discuss our assumptions and how they are justified by the current knowledge of how the competition is realized in these stages. Further, we discuss how the paternal outcrossing success differs among genotypes of faba bean and other plant species.

Our first assumption is that the maternal plant has a fixed share of self-fertilized seeds for a given set of paternal genotypes, thus only the remaining seeds originate from cross-fertilization. This assumption follows the framework and terminology of “mate choice” (Marshall et al. 1991), “maternal control” (Diggle et al. 2010) and “female choice” (Skogsmyr and Lankinen 2002; Mazer et al. 2010). A faba bean flower carries between two and ten ovules, but rarely develops more than five mature seeds while often embryos whose development stopped at an early stage are found (Bond and Poulsen 1983; Link and Stützel 1995; own observation), thus, a certain rate of embryo abortion is prevalent in faba bean. It has been shown that non-inbred embryos exhibit a stronger sink for assimilates, thus have a faster seed development and result in heavier seeds than inbred embryos (Duc and Rowland 1990; Meitzel et al. 2011). Thus, a self-fertilized embryo might have a lower chance of developing into a mature seed than an inter-genotype cross-fertilized (i.e. hybrid) embryo. The degree of cross-fertilization only takes the mature seeds into account. A higher abortion rate of self-fertilized embryos would contribute to the degree of cross-fertilization reflecting how maternal control directly influences the degree of cross-fertilization. However, the relative impacts of the maternal plant, the pollen donor and the embryo to the reproductive success are largely unclear (Marshall et al. 1991; Snow and Spira 1991b; Skogsmyr and Lankinen 2002; Bernasconi 2003) although they might have important implications for the degree of cross-fertilization in any given situation or scenario (Snow and Spira 1991a).

In diverse populations, the probability that an individual plant is cross-fertilized by pollen of other plants that are genetically identical is neglectable (Busbice 1969). Our experimental layout with 64 plants of each genotype per polycross, however, follows the common way of producing synthetics and results in the situation that pollen can be transferred between genetically identical plants, effectuating intra-genotype cross-fertilization. Following Geiger (1982), our second assumption is that the share of intra-genotype cross-fertilization is 1/8 of the cross-fertilized seeds of each of the eight maternal genotypes. To assume a consistent share of 1/8 among genotypes might overestimate the size of intra-genotype cross-fertilization for those genotypes with a low level of P, and underestimate it for genotypes with a high level of P. We conclude that attributing a consistent share of 1/8 intra-genotype cross-fertilization to all genotypes was a conservative approach while methods that allow to distinguish between intra-genotype pollen and self-pollen would result in larger differences of P between genotypes. Furthermore, in a polycross, synchronous flowering is achieved best between plants of the same genotype. Thus, due to an optimal synchrony of flowering, intra-genotype cross-pollen is likely to have an advantage over pollen from plants of a different genotype, resulting in a decreased degree of cross-fertilization in the polycross progeny. Generally, considerations of the size of intra-genotype cross-fertilization are most relevant in the Syn-1 generation and have a decreasing effect in the subsequent generations. In later synthetic generations, genotypes occur as unique individuals in populations, thus intra-genotype cross-fertilization is absent.

We found significant differences in paternal outcrossing success P between faba bean genotypes and a high variation in this trait. P of faba bean likely depends on reproductive traits such as pollen quantity and pollen quality. Because hybrid vigour mainly affects reproductive traits (Link and Stützel 1995), we expected that heterotic faba bean plants realize a higher value of P than inbred ones. Indeed, in sets A and B, the F1 hybrids resulted in a markedly and significantly larger value of P than the inbred lines. All inbred lines except one were found to be below 12.5%, with the lowest inbred line achieving only 4%. In contrast, the F1 hybrids ranged from 18.5 to 23.9% and thus achieved a P value much larger than the 12.5% that would be expected if there was no difference in P (Fig. 4b, c).

We also found that the value of P of a specific genotype depends on the environment which includes the other genotypes in the set, the pollinator populations, and the general environmental conditions such as soil, climate, and weather. For example, the largest variation in P was found for line S_217, which had the lowest value of any genotype in any of the sets (4% in set B, Fig. 4c) and the highest value of any inbred line in any of the sets (17% in set 0, Fig. 4a). As we controlled both the neighbourhood and the concurrence of full bloom for the seeds that we chose for analysis (see Supplementary Figs. 2, 4), the dramatic differences observed for some genotypes in P could not result from these environmental conditions.

The value of P of a specific genotype can depend on the other genotypes in the same set in several ways. First, since the pollen of the specific genotype competes with all other genotypes, its P value should be affected by the pollen donor success rates of the other genotypes. Second, since the other genotypes also serve as maternal plants, the P value of the specific genotype is expected to depend on the compatibility of its pollen with the other genotypes. Indeed, the dependency of P on the other genotypes is shown by a significant paternal genotype × maternal genotype (PG × MG) term in the analysis of deviance of the GLM (Table 3). Furthermore, the PG × MG interaction varies across sets and years (Supplementary Tables 9, 11, 13, 15). While some genotypes had a consistently low interaction value on another genotype (e.g. S_046 on Fam157 in sets 0 2014, set A 2015 and set A 2016), the interaction varies strongly for other genotypes (e.g. S_046 on S_085 with a large positive value in set A, 2015 and negative values in set A, 2016 and set 0). This high variation of interaction effects is reflected in the significant deviance value for PG × MG × Year in set A (Table 3). It thus seems that some paternal and maternal genotypes are more compatible with each other than other pairs and this compatibility is affected by the environment. However, our data does not allow to fully characterize the extent of compatibility and the causes for its variation, which is an interesting subject for future studies.

A third way in which the other genotypes in the same set can influence the P values is the presence or absence of F1 hybrids. As the values of P of all genotypes in one polycross sum up to 100%, the higher values of P of F1 hybrids entail lower values for the inbred lines. In sets A and B where F1 hybrids were included, the inbred lines had generally lower values of P than in set 0, where only inbred lines were included. In set 0, the eight inbred lines ranged from 7 to 17%, thus some of them realized a higher value of P than the inbred lines in sets A and B, and none of them came near to the lowest of the inbred lines in sets A and B. Thus, a heterotic component was clearly present. In contrast to the trend of decreased values of P in the presence of F1 hybrids, line S_046 had higher P values in set A (13–14%, see Supplementary Table 7) than in set 0 (10%). Line S_085 had similar values in sets 0 and A (11.3–12.2%) but a lower value in set B (8.4%). Thus, the P value of an inbred line was generally lower in the presence of F1 hybrids, but again, there seem to be additional influences by other environmental factors.

Other environmental conditions apart from the surrounding genotypes that supposedly effect P are the pollinator populations and the general conditions such as soil, climate, and weather. We found some differences in genotypic responses to environmental conditions. For example, set B was grown at a location with shallow soil on limestone in slightly higher altitude than sets 0 and A, which were grown on deep soils with a good water storage capacity. It appears that, in this experiment, P of S_217 depended strongly on the growing conditions, with a low P value on non-optimal soil and a high P on good soil. The effects of site-specific stresses such as heat or shortage of water or nutrients might affect the pollen quality or the amount or composition of nectar and thus the attractiveness of genotypes to pollinators (Bailes et al. 2018). Besides genotypes responding differentially to the local situation, differential local pollinator populations might also react differentially to the specific attractiveness of these genotypes. The relevance of such environmental effects is supported by the significant deviance values of PG × Year (Table 3). We conclude that P values depend on environmental influences in various ways. Our data do not allow to differentiate between these various environmental influences. To quantify their effects on P, further studies are needed.

The paternal outcrossing success of faba bean is expected to be the combined result of pollen quantity, pollen quality and pollen dispersal. Whereas pollen dispersal depends on pollinator-related properties of a plant (attractiveness, transportability of the pollen) as well as on the small-scaled behaviour of the pollinators (see above), pollen quantity and pollen quality are likely showing heterosis or its complement, inbreeding depression. For faba bean, it has been previously shown that F1 hybrids produced higher pollen quantities than their parental inbred lines (Kambal et al. 1976; Chen 2009). F1 hybrids may as well show higher pollen quality (e.g. pollen grain size, pollen germination rate and pollen tube growth rate) than inbred lines. Some support to this hypothesis in the case of faba bean is given by Drayner (1959), who found that on agar media, pollen tubes from hybrid plants’ pollen grew faster than from two inbred lines. Another influence on P might be pollen tube growth rates (Snow and Spira 1991a, b; Skogsmyr and Lankinen 1999). Thus, hybrid plants might possess an advantage over inbred plants in terms of effectuating fertilization under natural conditions. Further investigations would be required to test these hypotheses for faba bean.

Differential paternal outcrossing success P has been found in other plants, too. In their study on insect-pollinated wild radish (*Raphanus sativus*), Marshall et al. (2000) found that the number of seeds sired per fruit depended on the maternal plant, the pollen donor and the interaction between both. As wild radish has sporophytic self-incompatibility, the seeds they considered for scoring paternal outcrossing success were all cross-fertilized, as were the seeds in our study when focussing on P. Marshall et al. (2000) also found large effects of the pollen donor on paternal outcrossing success and a minor effect of the interaction between maternal plant and pollen donor.

In several other plant species, studies on seed paternity have shown differences between genotypes in paternal outcrossing success, e.g. in autotetraploid alfalfa (*Medicago sativa* L.) (Riday et al. 2013), the insect-pollinated common morning glory (*Ipomoea purpurea*) (McCallum and Chang 2016), in the insect-pollinated annual plant *Arenaria uniflora* (Fishman 2000), in the neotropical vine *Dalechampia scandens* (Pélabon et al. 2015), as well as in tree species such as the wind pollinated Scots pine (*Pinus sylvestris*) (Torimaru et al. 2012), in Douglas fir (*Pseudotsuga menziesii*) (Apsit et al. 1989), and in the neotropical tree *Ceropia obtusifolia* (Kaufman et al. 1998), also see reviews by Bernasconi (2003) and Pannell and Labouche (2013).

The differences of P for different faba bean genotypes may be associated with the differential pollen quantity. For several other plant species, a positive correlation between the amount of exported pollen and the proportion of seeds sired has been confirmed (see Delph and Ashman 2006) and the amount of pollen produced has been shown as major explanatory factor for paternal outcrossing success in Scots pine (Torimaru et al. 2012).

### Relevance for breeding and seed production

Breeders work under the assumption that cross-pollen transmitted via pollinators originates in equal shares from all individual plants of a crop stand (Link et al. 1994a), which is relevant when employing the concept of combining ability. We found marked differences between the paternal outcrossing successes of different faba bean genotypes (Table 3, Fig. 4).

We found a highly negative correlation between degree of cross-fertilization and paternal outcrossing success. The size of correlation seems to depend on three aspects. First, there was a heterotic effect involved. In the sets A and B, the correlation was higher with all eight genotypes included than with inbred lines only. Second, it depended on the actual genotypes: the eight inbred lines of set 0 showed a higher negative correlation between the two traits than the six lines in each set A and B. Third, it may depend on the environment. The correlation between traits was high for the inbred lines of set A in 2016, but only medium in 2015.

With the assumption of a linear relationship between yield potential and inbreeding coefficient F (Geiger 1982), the development of both features across Syn-generations can be described (Wright 1922; Kinman and Sprague 1945; Busbice 1970; Geiger 1982; Link and Ederer 1993). In partially allogamous synthetic populations, the mean inbreeding coefficient will decrease and thus performance will increase from $F=1$ at Syn-0 to its equilibrium level which is approximately reached after few generations, earlier with higher values of C (Link et al. 1994a). In case of no variation for C and for P and further idealized conditions such as $N=\infty$ for the number of components, this value decreases from ${F}_{\mathrm{Syn}-0}=1$ and ${F}_{\mathrm{Syn}-1}=1-\mathrm{C}$ downwards and is ultimately steering towards its minimum value in Syn-∞, ${F}_{Syn-\infty }=(1-\text{C})/(1+\text{C})$ (Busbice 1969). It is thus relatively obvious how the inbreeding level F is affected by changes and variation of C, e.g. due to environment, genotype or the negative heterosis for the trait C itself. Higher figures for C result in smaller inbreeding, thus higher shares of heterosis, and therefore higher performance. The impact of variation in P on inbreeding and performance is less obvious.

To exemplify the possible impact of P, we discuss two extreme scenarios of synthetics, scenario one with equal P for all lines and scenario two with maximal differences of P values. In both scenarios, the synthetics consist of eight homozygous components as Syn-0, C is constantly 50%, and self-fertilization is realized without limitations. In scenario one, 50% of the individuals in Syn-1 originate from actual self-fertilization in Syn-0, and 1/8 of the 50% of the cross-fertilization is intra-genotypic cross-fertilization, which is genetically selfing. In this case, the inbreeding coefficient in generation Syn-1 is ${F}_{Syn-1}=$ 0.5625. In scenario two, we consider that P is zero for all lines except one, which hence is the only donor of cross-pollen. Self-pollen is available in each line without limitations. The seven lines with zero P have zero intra-genotypic cross-fertilization and fully realize their 50% of cross-fertilization with the only successfully siring line. The siring line does not find any cross-pollen and thus only and fully self-fertilizes, reducing the average value of C by 1/8, which is the same value as in scenario one. Consequently, the inbreeding coefficient in Syn-1 of this scenario is the same as in scenario one. However, the composition in Syn-2 is different in the scenarios. In scenario one, inbreeding decreases from ${F}_{\mathrm{Syn}-1}=$ 0.5625 to ${F}_{\mathrm{Syn}-2}=$ 0.4531. In scenario two with its dramatic difference in P, we arrive at ${F}_{\mathrm{Syn}-2}=$ 0.5967 given a recessive inheritance of zero paternal outcrossing success. Thus, scenario two results in an increased value for *F* in Syn-2 and all following synthetic generations will be impacted accordingly. As seen from these extreme scenarios, larger differences in P result in a markedly higher inbreeding coefficient from Syn-2 onwards.

In general, as P is different between the components, genetic diversity in the emerging synthetic cultivar is reduced, resulting in higher inbreeding in generations Syn-2 onwards, which leads to a reduced genetic yield potential of faba bean synthetics compared to current-state predictions that do not take this effect into account.

In addition to the level of inbreeding in later synthetic generations, variation in P among components leads to differential contributions of the components to the genetic make-up of the Syn-generations following Syn-0. It is those pollen donors with higher P who contribute a higher share of their alleles to the subsequent generations than pollen donors with a lower P. Thus, the more successful pollen donors determine the traits of the future generations more than the others. Variation in P increases inbreeding and thus inbreeding depression in subsequent synthetic generations and furthermore creates a shift in the allele frequencies and hence trait expression in synthetic generations. Both, inbreeding and allele frequencies, impact the trait expression in a synthetic.

Differences in P are relevant for the breeding of synthetics of partially allogamous crops with a marked degree of cross-fertilization, e.g. alfalfa (*Medicago sativa* L.) with 55% cross-fertilization (Riday et al. 2013), oilseed rape (*Brassica napus*) with about 30% cross-fertilization (Becker et al. 1992), pigeonpea (*Cajanus cajan*) with about 20% cross-fertilization (Saxena et al. 1990) and sorghum (*Sorghum bicolor*) with a mean of 18% cross-fertilization (Barnaud et al. 2008). The differences in P are even more relevant for completely allogamous crops such as maize, rye, sunflower, alfalfa and forage grasses, as the size of cross-fertilization is larger, and thus, differential paternal outcrossing success has an even larger effect on the subsequent synthetic generation.

## Conclusions

In this study, we experimentally quantified size and variation of two parameters that contribute to the level of inbreeding and allele frequencies of faba bean populations: degree of cross-fertilization and paternal outcrossing success. We found marked and significant genetic variation in both parameters, both within and between groups of different inbreeding status (inbred lines and F1 hybrids). With our unprecedented approach, we were able to show that assumptions of equal shares as pollen donors in faba bean are overly simplistic and are not grounded on biological reality. Consequently, the genetic change from one synthetic generation to the next is more complex than previously assumed. For optimal breeding of synthetics of crops with appreciable differences in paternal outcrossing success, these differences need to be considered.

One possibility to avoid an increase in inbreeding due to the variation in paternal outcrossing success P is to create the Syn-1 not as a mixture of inbred lines, but as a mixture of manually produced F1 hybrids, e.g. based on a diallel mating design. By taking the same number of seeds from each parental combination, the P values of all parental combinations will be identical from Syn-0 to Syn-1. In addition, a differential expression of P in the subsequent generations will be mitigated. For example, if one out of eight inbred lines has a low pollen quality that will be less extreme in all F1 hybrids which have this line as a parent. If in one inbred line a high value for P is combined with, e.g. a high resistance, the alleles effectuating these two traits will mostly segregate independently from Syn-1 to Syn-2, thereby loosening such association. However, a diallel cross between, e.g. eight parental lines would encompass 28 crosses (reciprocal crosses neglected) and producing sufficient seed at seed-selling scale in Syn-4 in a crop with a low reproduction rate such as faba bean would imply very high effort if Syn-1 was created from manual crossed seed. As the environment affects the values for P in several ways, a less strong mitigation strategy is to sow the same Syn-0 in several environments and to later combine the harvested seeds in a Syn-1 bulk.

These proposals allow to mitigate the effects of differential P values. To better understand the effects of differential P on later synthetic generations, it would be necessary to extend the algebra of yield prediction for synthetics to include these differences. Such data and algebra would further allow the estimation of the uncertainties in the predictions and has the potential to increase the precision of such predictions.

## Supplementary Information

Below is the link to the electronic supplementary material.

Supplementary file1 (PDF 838 kb)

## Data Availability

The datasets employed in this study are available from the corresponding author on reasonable request.

## Code availability

The R code used during the analysis and the Python script to identify seed parents are available from the corresponding author on reasonable request.
