# Supplementary material for: Paternal outcrossing success differs among faba bean genotypes and impacts breeding of synthetic cultivars
Source: Theor Appl Genet. 2021 May 7;134(8):2411–27. doi: 10.1007/s00122-021-03832-z (PMC8277637; doi:10.1007/s00122-021-03832-z)
Supplement: Supplementary file 1 — Supplementary file1 (PDF 838 kb) [file 122_2021_3832_MOESM1_ESM.pdf]

Paternal outcrossing success differs among faba bean genotypes and impacts breeding of synthetic cultivars

Lisa Brünjes, Wolfgang Link

Supplementary Figures

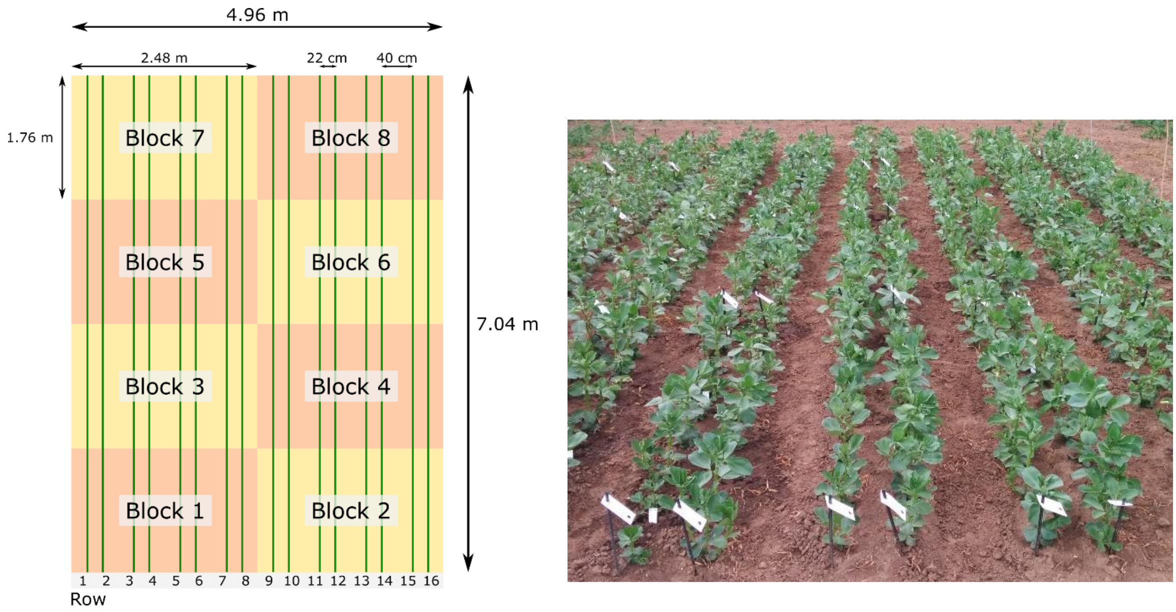

Supplementary Figure 1 Experimental design of a polycross with eight blocks and eight rows per block. The first seed of a row was sown 10 cm inside the row. The distance between the seeds within a row was 22 cm, resulting in a plant density of 15 single plants per m^2

Position \ Row	1	2	3	4	5	6	7	8	9	10	11	12	13	14	15	16
32	3	4	2	5	1	6	8	7	4	5	3	6	2	7	1	8
31	4	5	3	6	2	7	1	8	5	6	4	7	3	8	2	1
30	2	3	1	4	8	5	7	6	3	4	2	5	1	6	8	7
29	5	6	4	7	3	8	2	1	6	7	5	8	4	1	3	2
28	1	2	8	3	7	4	6	5	2	3	1	4	8	5	7	6
27	6	7	5	8	4	1	3	2	7	8	6	1	5	2	4	3
26	8	1	7	2	6	3	5	4	1	2	8	3	7	4	6	5
25	7	8	6	1	5	2	4	3	8	1	7	2	6	3	5	4
24	1	2	8	3	7	4	6	5	2	3	1	4	8	5	7	6
23	2	3	1	4	8	5	7	6	3	4	2	5	1	6	8	7
22	8	1	7	2	6	3	5	4	1	2	8	3	7	4	6	5
21	3	4	2	5	1	6	8	7	4	5	3	6	2	7	1	8
20	7	8	6	1	5	2	4	3	8	1	7	2	6	3	5	4
19	4	5	3	6	2	7	1	8	5	6	4	7	3	8	2	1
18	6	7	5	8	4	1	3	2	7	8	6	1	5	2	4	3
17	5	6	4	7	3	8	2	1	6	7	5	8	4	1	3	2
16	7	8	6	1	5	2	4	3	8	1	7	2	6	3	5	4
15	8	1	7	2	6	3	5	4	1	2	8	3	7	4	6	5
14	6	7	5	8	4	1	3	2	7	8	6	1	5	2	4	3
13	1	2	8	3	7	4	6	5	2	3	1	4	8	5	7	6
12	5	6	4	7	3	8	2	1	6	7	5	8	4	1	3	2
11	2	3	1	4	8	5	7	6	3	4	2	5	1	6	8	7
10	4	5	3	6	2	7	1	8	5	6	4	7	3	8	2	1
9	3	4	2	5	1	6	8	7	4	5	3	6	2	7	1	8
8	5	6	4	7	3	8	2	1	6	7	5	8	4	1	3	2
7	6	7	5	8	4	1	3	2	7	8	6	1	5	2	4	3
6	4	5	3	6	2	7	1	8	5	6	4	7	3	8	2	1
5	7	8	6	1	5	2	4	3	8	1	7	2	6	3	5	4
4	3	4	2	5	1	6	8	7	4	5	3	6	2	7	1	8
3	8	1	7	2	6	3	5	4	1	2	8	3	7	4	6	5
2	2	3	1	4	8	5	7	6	3	4	2	5	1	6	8	7
1	1	2	8	3	7	4	6	5	2	3	1	4	8	5	7	6

Supplementary Figure 2 Arrangement of entries (i.e. eight genotypes) in a polycross. The neighbourhood between different genotypes was completely balanced, see Morgan JP (1988) Polycross designs with complete neighbor balance, Euphytica 39: 59-63. The numbers 1 to 8 were randomly assigned to the eight genotypes in each set 0, A and B (for details, see Supplementary Tables 8 - 15)

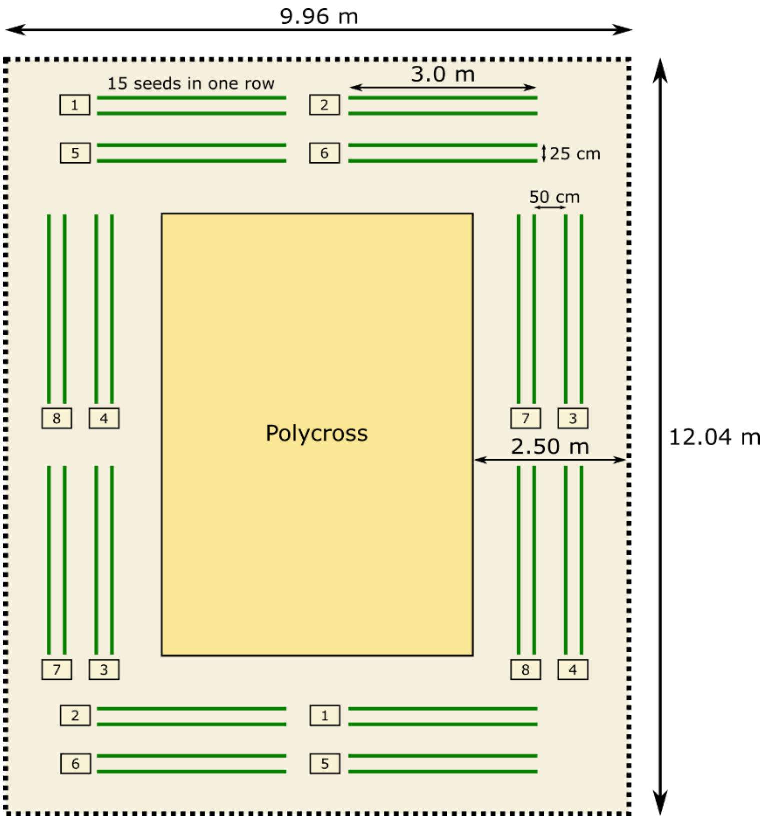

Supplementary Figure 3 Setup of the border rows surrounding each polycross. The numbers of the border rows refer to the respective entry number (i.e. the eight genotypes). The plants in the border rows were used as reserve in order to replace missing or sick single plants. They were removed at the onset of flowering

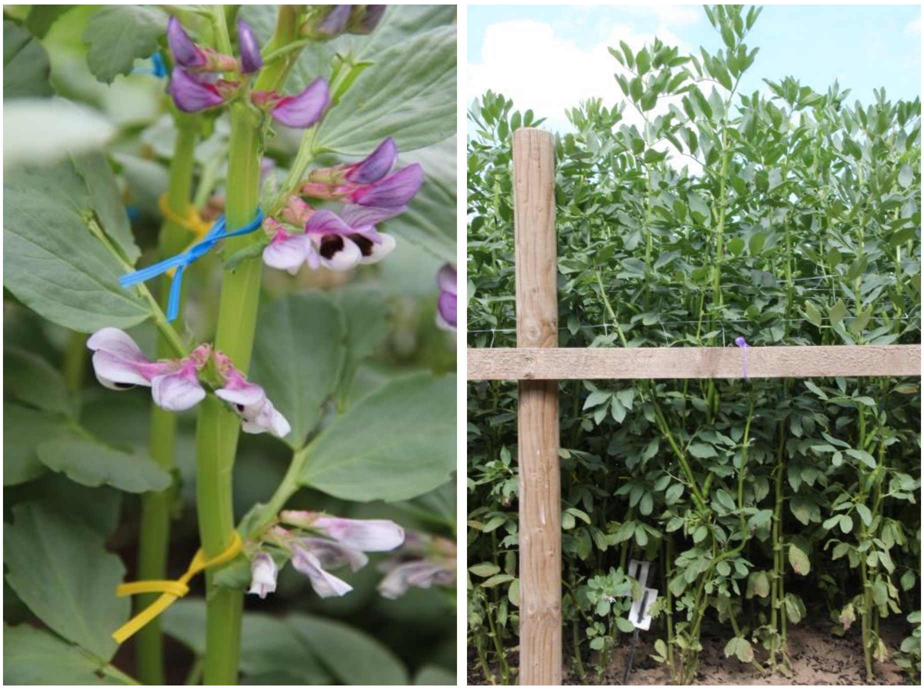

Supplementary Figure 4 Handling of plants during flowering. Left: Each tiller was labelled at the lowest (earliest) inflorescence (yellow label) and during concurrence of full bloom (CFB) of all genotypes at that specific inflorescence that was in full blossom on that particular day (blue label, see Supplementary Table 1). The pods that grew at the blue-labelled nodes were later chosen for SNP analysis. Right: Dense crop stand of winter faba bean. A wide-meshed net helped to prevent lodging of the plants

Supplementary Tables

Supplementary Table 1 Basic characteristics of the four studied polycrosses at three locations (GAR = Garteschänke, DRA = Dragoneranger, DEP = Deppoldshausen, S = sown in spring, W = sown in autumn). CFB = concurrence of full bloom

	Set 0, GAR 2014-S	Set A, DRA 2015-S	Set A, DRA 2016-S	Set B, DEP 2016-W
Sowing date	28.03.2014	20.03.2015	15.03.2016	02.10.2015
Replacement of sick or missing single plants	29.04.2014	08.05.2015	/	05.+11.04.2016
Number and percent of replaced single plants	13 (2.5%)	20 (3.9%)	0	39 (7.6%)
Begin of flowering	10.06.2014	10.06.2015	05.06.2016	11.05.2016
Labelling for CFB	2.-3.7.2014	15.-16.6.2015	12.06.2016	18.05.2016
Number and percent of single plants flowering at CFB	503 (98.2%)	511 (99.8%)	505 (98.6%)	501 (97.9%)

Supplementary Table 2 Number of seedlings with identified paternal genotype for each maternal genotype in Set 0, 2014, location Garteschänke. Total seedling number of the whole polycross, as well as minimum and maximum seedling number of the eight replicates are shown. Mean and variance are shown for the seedling numbers across the replicates

Maternal genotype	Total	Minimum	Maximum	Mean	Variance
**S_019**	511	13	98	63.88	1043.0
**S_025**	686	60	101	85.75	188.5
**S_035**	788	95	102	98.50	5.7
**S_046**	572	49	93	71.50	243.1
**S_085**	668	53	99	83.50	353.1
**S_120**	672	50	106	84.00	485.7
**S_217**	747	85	100	93.38	27.1
**Fam157**	718	78	99	89.75	45.1

Supplementary Table 3 Number of seedlings with identified paternal genotype for each maternal genotype in Set A, 2015, location Dragoneranger. Total seedling number of the whole polycross, as well as minimum and maximum seedling number of the eight replicates are shown. Mean and variance are shown for the seedling numbers across the replicates.

Maternal genotype	Total	Minimum	Maximum	Mean	Variance
**S_046**	778	81	108	97.25	76.2
**S_085**	735	72	107	91.88	160.4
**S_145**	697	59	101	87.13	151.8
**S_199**	754	78	109	94.25	147.1
**S_235**	839	97	116	104.88	42.1
**Fam157**	784	59	121	98.00	302.3
**F1(S_019 x S_035)**	791	91	111	98.88	52.1
**F1(S_025 x S_217)**	829	80	111	103.63	108.8

Supplementary Table 4 Number of seedlings with identified paternal genotype for each maternal genotype in Set A, 2016, location Dragoneranger. Total seedling number of the whole polycross, as well as minimum and maximum seedling number of the eight replicates are shown. Mean and variance are shown for the seedling numbers across the replicates

Maternal genotype	Total	Minimum	Maximum	Mean	Variance
**S_046**	775	76	106	96.88	89.6
**S_085**	766	86	105	95.75	74.8
**S_145**	772	82	108	96.50	67.4
**S_199**	769	89	104	96.13	35.3
**S_235**	721	64	102	90.13	171.0
**Fam157**	739	85	103	92.38	56.0
**F1(S_019 x S_035)**	796	94	109	99.90	21.4
**F1(S_025 x S_217)**	711	26	111	88.88	763.6

Supplementary Table 5 Number of seedlings with identified paternal genotype for each maternal genotype in Set B, 2016, location Deppoldshausen. Total seedling number of the whole polycross, as well as minimum and maximum seedling number of the eight replicates are shown. Mean and variance are shown for the seedling numbers across the replicates.

Genotype	Total	Minimum	Maximum	Mean	Variance
**S_003**	830	100	106	103.75	5.4
**S_019**	770	83	105	96.25	85.9
**S_025**	757	85	103	94.63	30.6
**S_035**	777	92	106	97.13	34.1
**S_085**	775	64	120	96.88	350.7
**S_217**	806	90	109	100.75	53.4
**F1(S_046 x S_085)**	792	73	107	99.00	135.7
**F1(Fam157 x S_199)**	805	93	111	100.63	40.8

Supplementary Table 6 Least square means of degree of cross-fertilization [%] and corresponding confidence intervals of sets 0, A and B in each environment. GAR = location Garteschänke, DRA = location Dragoneranger, DEP = location Deppoldshausen. C = Degree of cross-fertilization, UCI = Upper confidence interval, LCI = Lower confidence interval. Colours indicate the highest value (dark green) and the lowest value (dark red) in each column, thereby excluding the environment main-effect and showing trends across columns

**Genotype**	**Set 0**		**Set A**				**Set B**	
	GAR<br>2014		DRA<br>2015		DRA<br>2016		DEP<br>2016	
	C [%]	UCI<br>LCI	C [%]	UCI<br>LCI	C [%]	UCI<br>LCI	C [%]	UCI<br>LCI
S_046	54.74	60.4<br>49.0	38.65	43.9<br>33.6	48.46	53.8<br>43.2		
S_085	38.04	43.3<br>33.0	37.65	43.1<br>32.5	56.65	61.9<br>51.3	42.04	47.0<br>37.3
F1(S_046 x S_085)							17.59	21.6<br>14.2
S_199			37.65	43.0<br>32.6	50.07	55.4<br>44.7		
Fam157	28.27	33.1<br>23.9	35.83	41.1<br>30.9	57.92	63.2<br>52.5		
F1(S_199 x Fam157)							16.44	20.3<br>13.2
S_019	62.52	68.2<br>56.4					39.81	44.7<br>35.1
S_035	32.99	37.7<br>28.6					69.37	73.7<br>64.7
F1(S_019 x S_035)			25.09	29.9<br>20.8	37.76	43.0<br>32.8		
S_025	33.21	38.3<br>28.5					35.09	40.0<br>30.5
S_217	39.57	44.6<br>34.8					37.81	42.6<br>33.3
F1(S_025 x S_217)			13.32	17.2<br>10.2	31.61	37.0<br>26.7		
S_120	57.50	62.6<br>52.2						
S_145	-		52.46	58.0<br>46.8	54.70	59.9<br>49.3		
S_235	-		42.54	47.7<br>37.6	55.92	61.3<br>50.4		
S_003	-		-		-		48.19	53.0<br>43.5

Supplementary Table 7 Least square means of paternal outcrossing success [%] and corresponding confidence intervals of sets 0, A and B in each environment. GAR = location Garteschänke, DRA = location Dragoneranger, DEP = location Deppoldshausen. P = Paternal outcrossing success, UCI = Upper confidence interval, LCI = Lower confidence interval. Colours indicate the highest value (dark green) and the lowest value (dark red) in each column, thereby excluding the environment main-effect and showing trends across columns

Genotype	Set 0		Set A				Set B	
	GAR 2014		DRA 2015		DRA 2016		DEP 2016	
	P [%]	UCI LCI	P [%]	UCI LCI	P [%]	UCI LCI	P [%]	UCI LCI
S_046	10.18	12.0 8.6	13.45	15.8 11.4	14.07	16.0 12.4	-	
S_085	12.17	14.1 10.4	11.57	13.8 9.7	11.29	13.1 9.7	8.39	10.4 6.8
F1(S_046 x S_085)	-		-		-		19.58	22.0 17.3
S_199	-		5.62	7.4 4.3	11.59	13.4 10.0	-	
Fam157	11.35	13.2 9.7	8.22	10.2 6.6	5.05	6.4 4.0	-	
F1(S_199 x Fam157)	-		-		-		23.89	26.6 21.4
S_019	7.17	8.8 5.8	-		-		9.68	11.6 8.0
S_035	15.83	18.0 13.9	-		-		5.91	7.6 4.6
F1(S_019 x S_035)	-		22.22	25.0 19.6	24.88	27.3 22.6	-	
S_025	14.75	16.9 12.9	-		-		12.32	14.4 10.5
S_217	16.50	18.7 14.5	-		-		3.91	5.4 2.8
F1(S_025 x S_217)	-		21.13	24.0 18.5	16.20	18.2 14.4	-	
S_120	9.46	11.2 8.0	-		-		-	
S_145	-		6.13	8.4 4.4	7.69	9.2 6.4	-	
S_235	-		6.64	8.5 5.2	6.24	7.7 5.0	-	
S_003	-		-		-		11.14	13.1 9.4
**Mean**	**12.18**		**11.87**		**12.13**		**11.85**	

Supplementary Table 8 Values for P_{ij} [%] of each maternal genotype × paternal genotype combination as estimated least square means from the model in set 0, GAR 2014. The arithmetic means i and j are the mean of each MG or PG across the eight paternal or maternal genotypes. Each main effect j is calculated by subtracting the overall mean from the respective arithmetic mean of a paternal genotype. The overall mean is underlined. In addition to the arithmetic mean j, the least square (LS) mean j estimated directly for each paternal genotype is shown as a comparison.

		Paternal genotype j								
	Entry	1	2	3	4	5	6	7	8	
	Genotype	S_046	S_085	Fam157	S_025	S_217	S_019	S_035	S_120	Arith. mean i
Maternal genotype i	S_046	12.32	13.45	12.04	19.05	12.32	7.84	16.81	6.16	12.5
	S_085	8.25	12.71	14.78	9.28	18.56	8.93	16.49	11.00	12.5
	Fam157	8.23	13.42	12.12	19.05	16.02	5.19	12.12	13.85	12.5
	S_025	17.76	10.42	12.36	11.97	19.31	4.63	13.9	9.65	12.5
	S_217	7.10	15.38	11.83	14.79	12.43	7.99	20.71	9.76	12.5
	S_019	9.02	11.48	8.74	16.67	13.11	12.57	19.13	9.29	12.5
	S_035	17.79	14.43	9.06	12.42	18.79	8.72	12.75	6.04	12.5
	S_120	6.35	7.71	10.88	17.46	24.26	4.54	16.33	12.47	12.5
Arithmetic mean j		10.85	12.38	11.48	15.08	16.85	7.55	16.03	9.78	12.5
Main effect j		-1.65	-0.12	-1.02	2.58	4.35	-4.95	3.53	-2.72	0
LS mean j		10.18	12.17	11.35	14.75	16.50	7.17	15.83	9.46	11.82

Supplementary Table 9 Interaction effect between each maternal genotype (MG) × paternal genotype (PG) combination on P [%] in set 0, GAR 2014. The interaction effect is calculated as the value for P_{ij} from Supplementary Table 8 minus the overall mean minus the main effect j. For example, the value for P_{ij} of PG S_085 with MG S_046 was 13.45, hence the interaction effect of PG S_085 with MG S_046 is 13.45-12.5-(-0.12) = 1.07. The maximum and minimum value of each column are given in bold numbers.

		Paternal genotype j								
	Entry	1	2	3	4	5	6	7	8	
	Genotype	S_046	S_085	Fam157	S_025	S_217	S_019	S_035	S_120	Mean i
Maternal genotype i	S_046	1.47	1.07	0.57	**3.96**	**-4.53**	0.29	0.78	-3.62	0
	S_085	-2.60	0.34	**3.30**	**-5.81**	1.71	1.38	0.47	1.22	0
	Fam157	-2.63	1.04	0.64	**3.96**	-0.83	-2.36	**-3.91**	**4.07**	0
	S_025	6.91	-1.95	0.88	-3.12	2.46	-2.92	-2.13	-0.13	0
	S_217	-3.75	**3.01**	0.36	-0.29	-4.42	0.44	**4.68**	-0.02	0
	S_019	-1.83	-0.90	**-2.73**	1.58	-3.74	**5.02**	3.10	-0.49	0
	S_035	**6.93**	2.05	-2.42	-2.67	1.94	1.17	-3.28	**-3.74**	0
	S_120	**-4.50**	**-4.67**	-0.59	2.38	**7.41**	**-3.02**	0.30	2.69	0
Mean j		0	0	0	0	0	0	0	0	

Supplementary Table 10 Values for P_{ij} [%] of each maternal genotype × paternal genotype combination as estimated least square means from the model in set A, DRA 2015. The arithmetic means i and j are the mean of each MG or PG across the eight paternal or maternal genotypes. Each main effect j is calculated by subtracting the overall mean from the respective arithmetic mean of a paternal genotype. The overall mean is underlined. In addition to the arithmetic mean j, the least square (LS) mean j estimated directly for each paternal genotype is shown as a comparison.

		Paternal genotype j								
	Entry	1	2	3	4	5	6	7	8	
	Genotype	S_085	S_046	S_235	F1 (S_019 x S_035)	F1 (S_025 x S_217)	Fam157	S_145	S_199	Arith. mean i
Maternal genotype i	S_085	12.06	17.14	6.03	27.94	18.10	7.30	7.30	4.13	12.5
	S_046	13.70	12.54	6.71	24.49	16.91	10.79	9.62	5.25	12.5
	S_235	13.48	10.54	12.50	22.30	24.26	8.09	4.66	4.17	12.5
	F1 (S_019 x S_035)	12.28	15.79	3.95	12.28	34.65	4.39	12.72	3.95	12.5
	F1 (S_025 x S_217)	8.59	14.06	12.50	36.72	12.50	10.16	1.56	3.91	12.5
	Fam157	12.73	11.49	4.66	19.25	27.02	12.42	3.73	8.70	12.5
	S_145	10.79	14.15	2.40	28.30	21.10	4.32	12.71	6.24	12.5
	S_199	9.85	12.92	12.31	13.85	19.69	12.92	5.85	12.62	12.5
Arith. mean j		11.69	13.58	7.63	23.14	21.78	8.80	7.27	6.12	<u>12.5</u>
Main effect j		-0.81	1.08	-4.87	10.64	9.28	-3.70	-5.23	-6.38	0
LS mean j		11.57	13.45	6.64	22.22	21.13	8.22	6.13	5.62	10.58

Supplementary Table 11 Interaction effect between each maternal genotype (MG) × paternal genotype (PG) combination on P [%] in set A, DRA 2015. The interaction effect is calculated as the value for P_{ij} from Supplementary Table 10 minus the overall mean minus the main effect j. For example, the value for P_{ij} of PG S_046 with MG S_085 was 17.14, hence the interaction effect of PG S_046 with MG S_085 is 17.14-12.5-1.08 = 3.56. The maximum and minimum value of each column are given in bold numbers.

		Paternal genotype j								
	Entry	1	2	3	4	5	6	7	8	
	Genotype	S_085	S_046	S_235	F1(S_019 x S_035)	F1(S_025 x S_217)	Fam157	S_145	S_199	Mean i
Maternal genotype i	S_085	0.38	**3.56**	-1.60	4.80	-3.68	-1.50	0.03	-1.99	0
	S_046	**2.02**	-1.04	-0.93	1.35	-4.87	1.99	2.35	-0.87	0
	S_235	1.79	**-3.04**	**4.87**	-0.84	2.49	-0.71	-2.61	-1.95	0
	F1 (S_019 x S_035)	0.59	2.21	-3.68	**-10.86**	**12.87**	-4.41	**5.45**	-2.17	0
	F1 (S_025 x S_217)	**-3.09**	0.48	**4.87**	**13.58**	**-9.28**	1.36	**-5.71**	**-2.21**	0
	Fam157	1.05	-2.09	-2.97	-3.89	5.24	3.62	-3.54	2.58	0
	S_145	-0.90	0.57	**-5.23**	5.16	-0.68	**-4.48**	5.44	0.12	0
	S_199	-1.84	-0.66	4.68	-9.29	-2.09	**4.13**	-1.42	**6.50**	0
Mean j		0	0	0	0	0	0	0	0	

Supplementary Table 12 Values for P_{ij} [%] of each maternal genotype × paternal genotype combination as estimated least square means from the model in set A, DRA 2016. The arithmetic means i and j are the mean of each MG or PG across the eight paternal or maternal genotypes. Each main effect j is calculated by subtracting the overall mean from the respective arithmetic mean of a paternal genotype. The overall mean is underlined. In addition to the arithmetic mean j, the least square (LS) mean j estimated directly for each paternal genotype is shown as a comparison.

		Paternal genotype j								
	Entry	1	2	3	4	5	6	7	8	
	Genotype	S_085	S_046	S_235	F1(S_019 x S_035)	F1(S_025 x S_217)	Fam157	S_145	S_199	Arith. mean i
Maternal genotype i	S_085	12.50	13.31	6.85	27.42	14.52	4.64	5.65	15.12	12.5
	S_046	8.80	12.50	7.41	24.07	15.51	5.09	10.19	16.44	12.5
	S_235	11.71	17.57	12.58	22.34	14.97	3.90	6.07	10.85	12.5
	F1(S_019 x S_035)	14.87	16.33	7.58	12.24	24.49	5.25	7.87	11.37	12.5
	F1(S_025 x S_217)	9.69	15.12	3.88	39.15	12.40	5.04	6.98	7.75	12.5
	Fam157	9.63	9.43	5.74	23.98	19.26	12.30	7.38	12.30	12.5
	S_145	10.74	17.36	3.1	33.68	11.57	2.48	12.4	8.68	12.5
	S_199	13.57	12.67	6.56	22.62	19.91	5.43	6.79	12.44	12.5
Arith. mean j		11.44	14.28	6.71	25.69	16.58	5.52	7.91	11.87	12.5
Main effect j		-1.06	1.78	-5.79	13.19	4.08	-6.98	-4.59	-0.63	0
LS mean j		11.29	14.07	6.24	24.88	16.20	5.05	7.69	11.59	10.93

Supplementary Table 13 Interaction effect between each maternal genotype (MG) × paternal genotype (PG) combination on P [%] in set A, DRA 2016. The interaction effect is calculated as the value for P_{ij} from Supplementary Table 12 minus the overall mean minus the main effect j. For example, the value for P_{ij} of PG S_046 with MG S_235 was 17.57, hence the interaction effect of PG S_046 with MG S_235 is 17.57-12.5-1.78 = 3.29. The maximum and minimum value of each column are given in bold numbers.

		Paternal genotype j								
	Entry	1	2	3	4	5	6	7	8	
	Genotype	S_085	S_046	S_235	F1(S_019 x S_035)	F1(S_025 x S_217)	Fam157	S_145	S_199	Mean i
Maternal genotype i	S_085	1.06	-0.98	0.14	1.73	-2.06	-0.88	**-2.27**	3.25	0
	S_046	**-2.64**	-1.78	0.70	-1.61	-1.07	-0.42	2.27	**4.57**	0
	S_235	0.27	**3.29**	**5.87**	-3.35	-1.61	-1.61	-1.84	-1.02	0
	F1(S_019 x S_035)	**3.43**	2.04	0.87	**-13.44**	**7.91**	-0.27	-0.04	-0.50	0
	F1(S_025 x S_217)	-1.75	0.83	-2.84	**13.46**	-4.18	-0.48	-0.94	**-4.12**	0
	Fam157	-1.81	**-4.86**	-0.97	-1.71	2.68	**6.78**	-0.54	0.43	0
	S_145	-0.70	3.07	**-3.61**	7.99	**-5.01**	**-3.04**	**4.48**	-3.19	0
	S_199	2.13	-1.61	-0.15	-3.06	3.33	-0.09	-1.13	0.58	0
Mean j		0	0	0	0	0	0	0	0	

Supplementary Table 14 Values for P_{ij} [%] of each maternal genotype × paternal genotype combination as estimated least square means from the model in set B, DEP 2016. The arithmetic means i and j are the mean of each MG or PG across the eight paternal or maternal genotypes. Each main effect j is calculated by subtracting the overall mean from the respective arithmetic mean of a paternal genotype. The overall mean is underlined. In addition to the arithmetic mean j, the least square (LS) mean j estimated directly for each paternal genotype is shown as a comparison.

		Paternal genotype j								
	Entry	1	2	3	4	5	6	7	8	
	Genotype	S_217	S_035	S_085	S_025	F1(S_046 x S_085)	S_003	F1 (Fam157 x S_199)	S_019	Arith. Mean i
Maternal genotype i	S_217	12.61	6.45	5.57	11.14	18.48	10.85	25.51	9.38	12.5
	S_035	4.28	12.66	5.26	12.83	19.90	6.91	25.00	13.16	12.5
	S_085	4.31	6.20	12.4	7.82	20.49	9.16	28.30	11.32	12.5
	S_025	1.02	4.75	6.44	12.54	24.75	20.00	23.39	7.12	12.5
	F1(S_046 x S_085)	3.18	2.55	29.3	9.55	12.74	8.28	27.39	7.01	12.5
	S_003	4.68	4.01	8.91	20.49	16.7	12.69	24.94	7.57	12.5
	F1 (Fam157 x S_199)	4.17	9.03	3.47	12.50	31.25	15.28	13.19	11.11	12.5
	S_019	3.49	6.40	9.59	14.83	16.28	10.17	26.45	12.79	12.5
Arith. mean j		4.72	6.51	10.12	12.71	20.07	11.67	24.27	9.93	12.5
Main effect j		-7.78	-5.99	-2.38	0.21	7.57	-0.83	11.77	-2.57	0
LS mean j		3.91	5.91	8.39	12.32	19.58	11.14	23.89	9.68	10.43

Supplementary Table 15 Interaction effect between each maternal genotype (MG) × paternal genotype (PG) combination on P [%] in set B, DEP 2016. The interaction effect is calculated as the value for P_{ij} from Supplementary Table 14 minus the overall mean minus the main effect j. For example, the value for P_{ij} of PG S_235 with MG S_085 was 6.20, hence the interaction effect of PG S_235 with MG S_085 is 6.20-12.5-(-5.99) = -0.31. The maximum and minimum value of each column are given in bold numbers.

		Paternal genotype j								
	Entry	1	2	3	4	5	6	7	8	
	Genotype	S_217	S_035	S_085	S_025	F1(S_046 x S_085)	S_003	F1 (Fam157 x S_199)	S_019	Mean i
Maternal genotype i	S_217	**7.89**	-0.05	-4.55	-1.57	-1.60	-0.82	1.24	-0.55	0
	S_035	-0.44	**6.16**	-4.86	0.12	-0.17	**-4.76**	0.73	**3.23**	0
	S_085	-0.40	-0.31	2.28	**-4.90**	0.41	-2.50	**4.03**	1.39	0
	S_025	**-3.70**	-1.76	-3.68	-0.17	4.67	**8.33**	-0.88	-2.81	0
	F1(S_046 x S_085)	-1.53	**-3.96**	**19.18**	-3.16	**-7.33**	-3.39	3.12	**-2.93**	0
	S_003	-0.04	-2.50	-1.21	**7.78**	-3.37	1.03	0.67	-2.36	0
	F1 (Fam157 x S_199)	-0.55	2.52	**-6.65**	-0.21	**11.18**	3.61	**-11.08**	1.18	0
	S_019	-1.23	-0.11	-0.53	2.11	-3.79	-1.49	2.18	2.86	0
Mean j			0	0		0	0	0	0	
